# Uncovering the Fate and Risks of Intravenously Injected Prussian Blue Nanoparticles in mice by an Integrated Methodology of Toxicology, Pharmacokinetics, Proteomics, and Metabolomics

**DOI:** 10.1186/s12989-023-00529-7

**Published:** 2023-05-05

**Authors:** Haijing Qu, Xing Jin, Wei Cheng, Dongqi Wu, Boyu Ma, Chenmei Lou, Jian Zheng, Lijia Jing, Xiangdong Xue, Yang Wang

**Affiliations:** 1 School of Life Science, Northeast Forestry University, Harbin, 150040 China; 2 School of Pharmacy, Shanghai Frontiers Science Center for Drug Target Identification and Drug Delivery, Shanghai Jiao Tong University, Shanghai, 200240 China

**Keywords:** Prussian blue nanoparticles, Toxicology, Pharmacokinetics, Proteomics, Metabolomics

## Abstract

**Background:**

Prussian blue (PB) nanoparticles (NPs) have been intensively investigated for medical applications, but an in-depth toxicological investigation of PB NPs has not been implemented. In the present study, a comprehensive investigation of the fate and risks of PB NPs after intravenous administration was carried out by using a mouse model and an integrated methodology of pharmacokinetics, toxicology, proteomics, and metabolomics.

**Results:**

General toxicological studies demonstrated that intravenous administration of PB NPs at 5 or 10 mg/kg could not induce obvious toxicity in mice, while mice treated with a relatively high dose of PB NPs at 20 mg/kg exhibited loss of appetite and weight decrease in the first two days postinjection. Pharmacokinetic studies revealed that intravenously administered PB NPs (20 mg/kg) underwent fast clearance from blood, highly accumulated in the liver and lungs of mice, and finally cleared from tissues. By further integrated proteomics and metabolomics analysis, we found that protein expression and metabolite levels changed significantly in the liver and lungs of mice due to the high accumulation of PB NPs, leading to slight inflammatory responses and intracellular oxidative stress.

**Conclusions:**

Collectively, our integrated experimental data imply that the high accumulation of PB NPs may cause potential risks to the liver and lungs of mice, which will provide detailed references and guidance for further clinical application of PB NPs in the future.

**Supplementary Information:**

The online version contains supplementary material available at 10.1186/s12989-023-00529-7.

## Background

Nanomaterials (NMs) have attracted considerable interest in the biomedical field [1–4]. To date, a variety of NMs have been explored for medical applications such as imaging diagnosis, drug delivery, and novel therapeutics [5–8]. However, an increasing number of studies have revealed the potential biotoxicities of NMs [9–13]. Therefore, an in-depth understanding of the toxicity of candidate medical NMs is essential and very instructive for their further medical applications.

Prussian blue (PB) is a clinically approved oral drug for treating thallium and cesium poisoning [14]. Owing to their energy conservation and magnetic resonance properties, PB nanoparticles (PB NPs) have been used for photoacoustic/magnetic resonance imaging and photothermal therapies [15–18]. PB, as an oral antidote, can be excreted from feces and shows only a series of mild toxicities in patients, such as hypokalemia, constipation, and gastrointestinal discomfort [14, 19]. However, for imaging or therapy of specific lesions in vivo, PB NPs are usually administered by intravenous injection (i.v. ), which differs from oral administration in the pharmacokinetics (PK) profile and is more likely to induce toxic risks [9]. To date, research on the biosafety of PB NPs via i.v. administration was preliminarily focused on acute toxicity, short-term biodistribution, and histopathological changes, but these results are indistinct and need further elucidation [16–20]. Firstly, PB NPs with different modifications showed diverse toxicities after i.v. administration. For example, polyethylene glycol (PEG)-modified PB NPs are safe for mice, while polyvinylpyrrolidone (PVP)-modified PB NPs showed acute toxicity to the liver and the immune system of mice [15, 16, 19, 21]. This paradoxical result may be attributed to the different surface modifiers rather than the PB matrix. In addition, the accumulation and retention of NMs in tissues may affect local protein expression and metabolism, thereby inducing oxidative stress, the inflammatory response, and other toxicological risks [22–24]. Although several studies have shown that PB NPs can be gradually cleared by mouse bodies after i.v. administration [20, 21], the toxicological risks of PB NPs to exposed tissues during the intracorporal period have not been thoroughly elucidated to date.

In the present study, a comprehensive investigation of the fate and risks of PB NPs after i.v. administration was carried out by using a mouse model and an integrated methodology of pharmacokinetics, toxicology, proteomics, and metabolomics. Here, citric acid-capped PB NPs were chosen for our investigation. Citric acid is a nontoxic small molecule that can be metabolized by animals; thus, using citric acid as a capping agent for PB NPs can diminish the interference of surface modifiers in toxicity assays and more objectively uncover the toxicity of the PB matrix to the exposed tissues. A systematic PK study was used to investigate the blood clearance, biodistribution, and tissue clearance of PB NPs in mice. Integrated toxicology, proteomics, and metabolomics methodologies were used to obtain a comprehensive understanding of the risks of PB NPs to mice in terms of external symptoms, hematological and biochemical parameters, and histopathology after the injection of PB NPs, as well as to deeply uncover the response of highly exposed organs to PB NPs by integrated proteomics and metabolomics analysis. Our study might provide detailed knowledge to guide the clinical translational applications of PB NPs in the future.

**Fig. 1:**
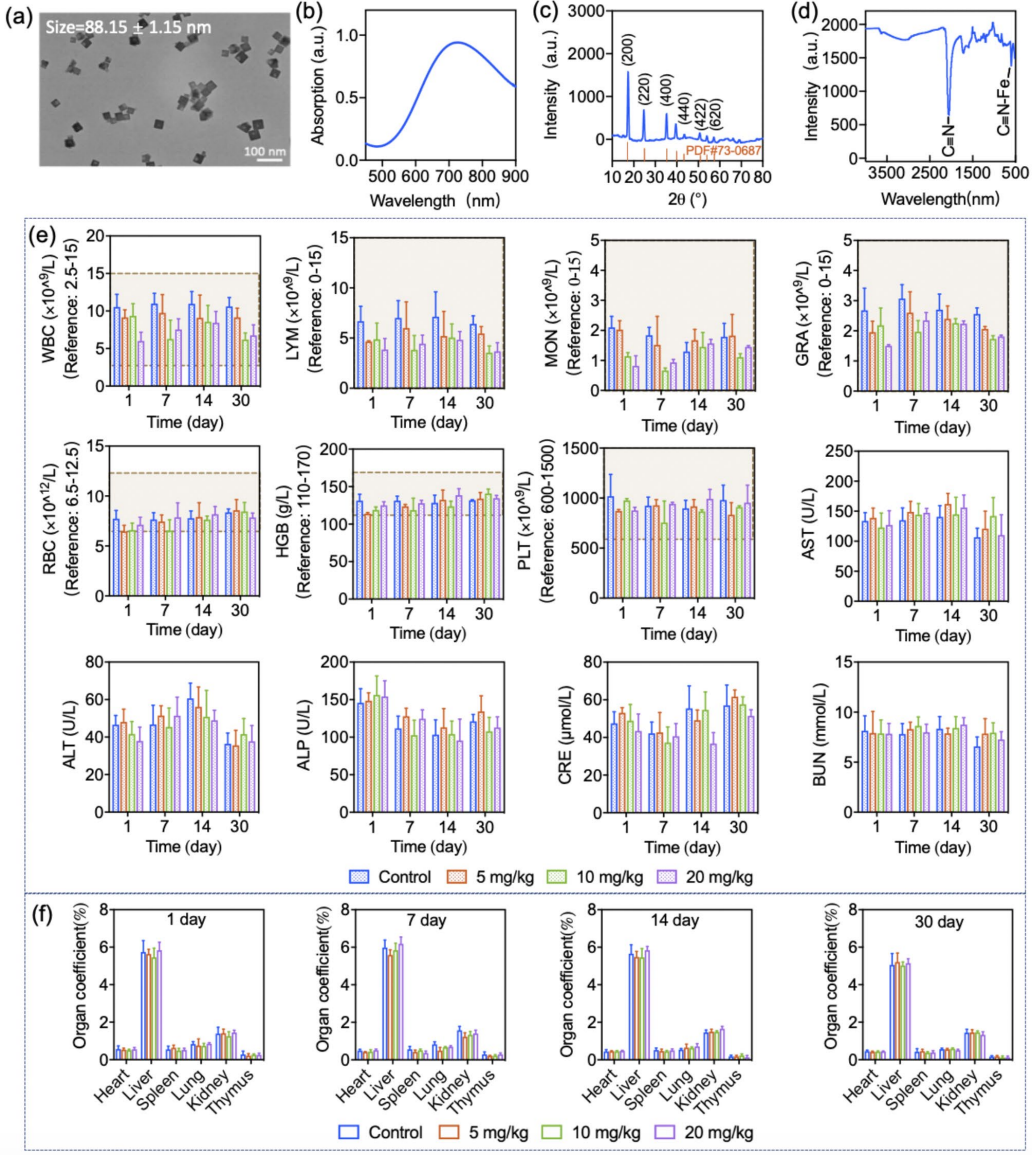
Characterization of PB NPs and toxicological evaluations of mice with different injections of PB NPs. (**a**) TEM micrograph of PB NPs. (**b**) UV‒Vis absorption of PB NPs. (**c**) XRD spectrum of PB NPs. (**d**) FTIR spectrum of PB NPs. (**e**) Hematological and biochemical parameters of mice treated with different doses of PB NPs. Annotation: WBC-white blood cell, MON-monocyte, GRA-granulocyte, LYM-lymphocyte, RBC-red blood cell, HGB-haemoglobin, PLT-platelet. Liver function parameters: ALP-alkaline phosphatase, AST-glutamic oxaloacetic transaminase, ALT-glutamic pyruvic transaminase. Kidney function parameters: BUN-blood urea nitrogen, CRE-creatinine. The red dashed line represents the normal range. (**f**) Organ coefficients of mice treated with different doses of PB NPs.

## Results

### Synthesis and characterization of PB NPs

Citric acid-capped PB NPs were fabricated by a hydrothermal synthetic method [25]. The hydrodynamic diameter and polydispersity (PDI) of PB NPs were measured by DLS as 88.15 ± 1.15 nm and 0.105 ± 0.015, respectively. The zeta potential of PB NPs was measured as -37.87 ± 1.72 mV. Under TEM observation, the PB NPs showed uniform cubic morphology with a practical diameter of 60 ~ 70 nm (Fig. 1a). The prepared PB NPs showed characteristic absorption in the near-infrared (NIR) region (Fig. 1b). PB NPs showed distinctive diffraction peaks under X-ray irradiation (2θ values of 17.45, 24.77, 35.30, 39.62, 43.68, 50.86, 54.07 and 56.87°) (Fig. 1c). In addition, distinct bands at 2084 cm^− 1^ and 498 cm^− 1^ that correspond to the vibration of the Fe^II^-CN-Fe^III^ and C ≡ N groups were observed in the FTIR spectrum of PB NPs (Fig. 1d). All the results indicated the successful fabrication of PB NPs.

### General toxicity studies

**Fig. 2:**
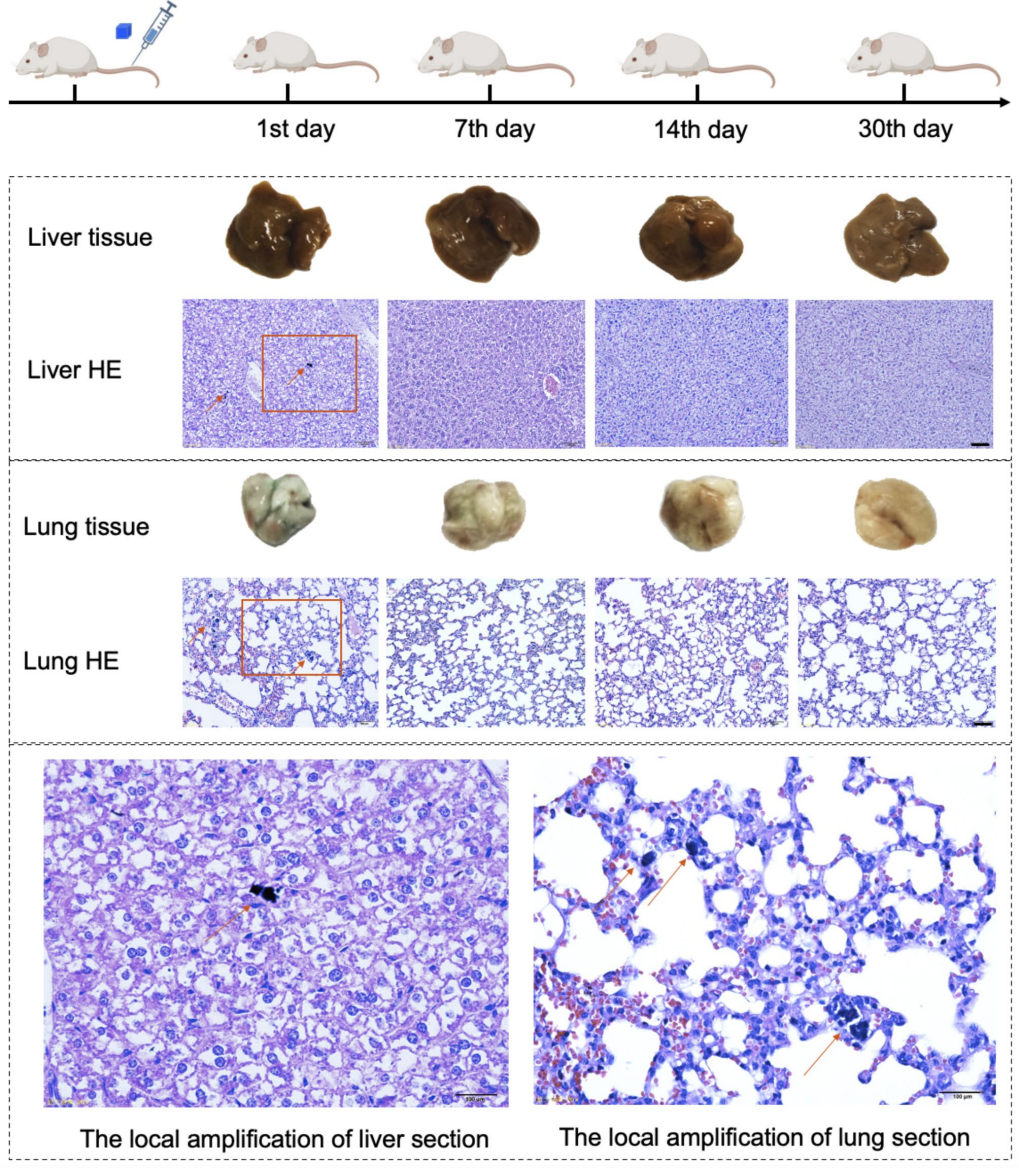
Histopathological sections of liver and lung on the 1st, 7th, 14th, and 30th day after intravenous injection of PB NPs at a 20 mg/kg dose. (Bar: 100 μm. Arrow: PB NPs)

General toxicity studies were performed after i.v. injection of PB NPs into mice at doses of 5, 10, and 20 mg/kg. The control group was i.v. injected with saline. No mouse death occurred in any of the treated groups during the experimental period. It was observed that different from other treated groups, the mice treated with 20 mg/kg of PB NPs exhibited dyspnea and bradykinesia at the initial stage postinjection, while the symptoms were temporary and could recover within 30 min. The body weight changes of mice were monitored as shown in Supplementary Fig. S1. For mice treated with 5 or 10 mg/kg PB NPs, the increase in body weight was similar to that of the saline group within 30 days, while mice treated with 20 mg/kg PB NPs showed obvious loss of weight during the initial 2 days, and the body weight subsequently increased similarly to that of the control group. Furthermore, we monitored the food intake of mice with different treatments. Compared with other treated groups, mice treated with 20 mg/kg PB NPs showed a loss of appetite and a decrease in food intake (Supplementary Fig. S2), which may account for the body weight loss in the initial 2 days. To further uncover the potential toxicity of PB NPs, evaluations of the hematological and biochemical parameters, organ coefficient indices, and histopathology were carried out. Compared with mice treated with saline and low doses of PB NPs (5 and 10 mg/kg), mice treated with 20 mg/kg PB NPs showed similar hematological and biochemical parameters (Fig. 1e). For mice treated with 20 mg/kg PB NPs on the 1st day postinjection, the livers and lungs presented a dark color, and PB NPs were observed distinctly in the liver and lung tissue sections, while this dark color gradually faded with time (Fig. 2). Although discernible high accumulation of PB NPs was found in the liver and lungs after injection, the coefficients and histopathology of the main organs showed no changes compared with the saline group and the other two treatment groups (Fig. 1f, Supplementary Fig. S3-S6).

### Pharmacokinetic Profile and Protein Corona Component of PB NPs

**Fig. 3:**
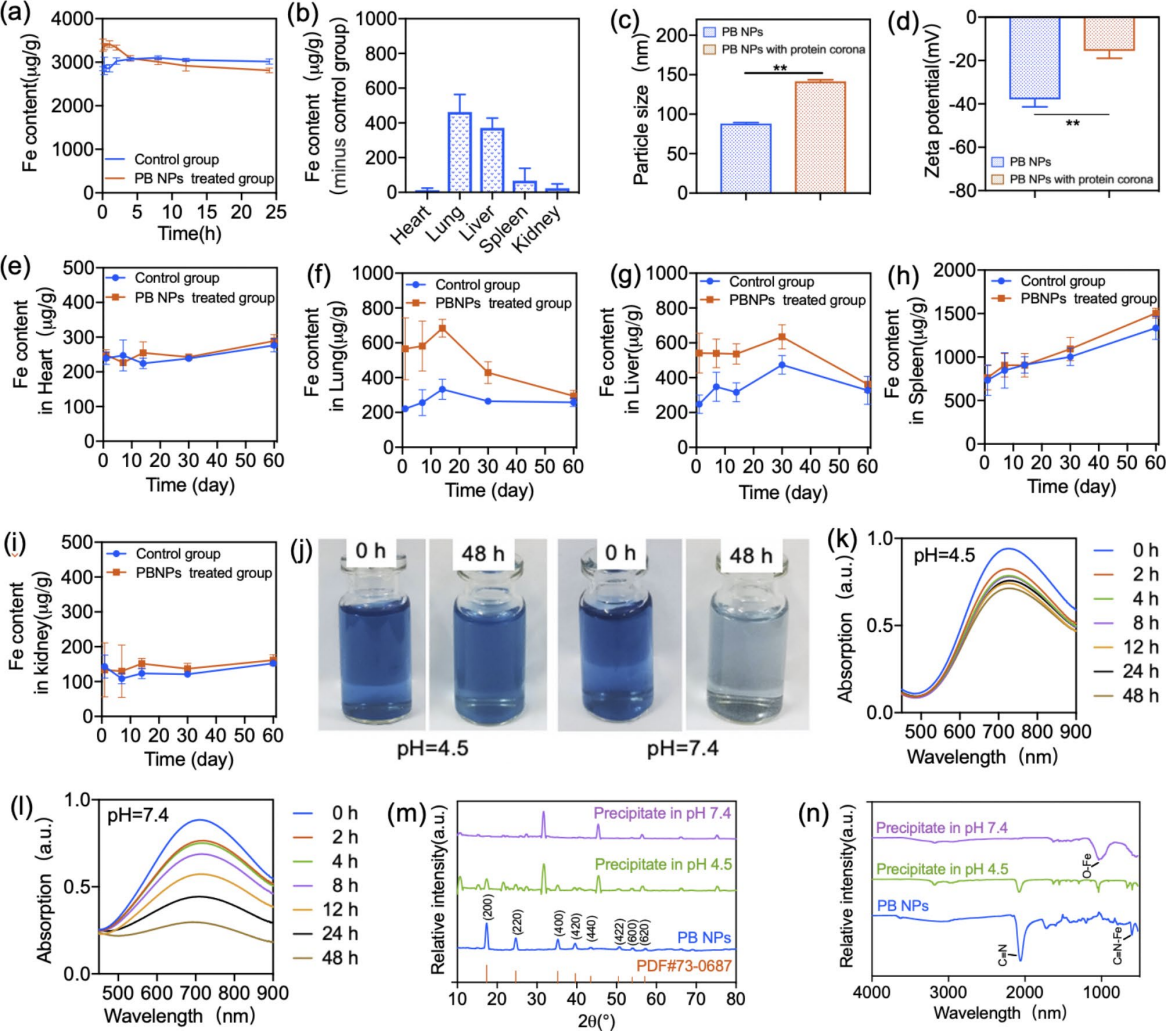
PK profile and degradation of PB NPs. (**a**) Changes in Fe content in the blood within 24 h after i.v. administration of 20 mg/kg PB NPs. (**b**) Changes in Fe content in major tissues at 24 h after i.v. administration of 20 mg/kg PB NPs. **(c)** The particle size of PB NPs with or without the protein corona. **(d)** Zeta potential of PB NPs with or without the protein corona. **(e-i)** The Fe content-time changes in the heart, lung, liver, spleen, and kidney after deducting the endogenous Fe content. **(j)** Changes in the color of PB NPs after incubation in SBF at pH 4.5 and pH 7.4. **(k)** and **(l)** Changes in the UV‒vis absorption of PB NPs after incubation in SBF at pH 4.5 and pH 7.4, respectively. **(m)** The XRD patterns and **(n)** FTIR spectra of PB NPs after incubation in SBFs at pH 7.4 and pH 4.5, respectively

PK investigation was performed to understand the fate of PB NPs in mice after i.v. injection. The blood clearance and biodistribution of PB NPs were determined by quantitative detection of the Fe content derived from PB NPs in the blood and the main organs via ICP‒OES after i.v. injection of PB NPs into mice at a dose of 20 mg/kg. As shown in Fig. 3a, compared with the control group, mice treated with PB NPs showed elevated blood Fe levels postinjection, while the high blood Fe level gradually decreased with time and was similar to that of the control group at 4 h postinjection, indicating that PB NPs undergo fast clearance from blood postinjection. By deducting the background blood Fe level, the PK parameters of Fe from PB NPs in the blood, including AUC_0-∞_, T_1/2_, and MRT, were calculated as 1365.89 ± 206.79 µg/g·h, 1.00 ± 0.41 h, and 1.70 ± 0.44 h, respectively (Supplementary table S1). The biodistribution of PB NPs was investigated by measuring the PB NP-derived Fe content in the tissues. For each tissue, the PB NP-derived Fe content was calculated by deducting the endogenous tissue Fe content from the total Fe content of the PB NP-containing tissue. For accurate detection of Fe content in tissues, the mouse tissues were perfused with saline to remove residual blood (Supplementary Fig. S7) to eliminate interference from endogenous Fe in the blood. As shown in Fig. 3b, by deducting the endogenous Fe content, the liver and lungs of treated mice showed obviously increased Fe content at 24 h after injection, while the heart, spleen, and kidneys did not exhibit increased Fe content. The results indicated that PB NPs mainly accumulated in the liver and lungs after fast clearance from the blood.

It was reported that the protein corona is a key factor that can affect the biodistribution of NPs [26–28]; thus, we investigated the formation and components of the protein corona of PB NPs after incubation with fresh mouse plasma. After incubation, the particle size of PB NPs increased to 141.6 ± 2.09 nm, and the zeta potential of PB NPs decreased to -15.56 ± 3.42 mV (Fig. 3c and 3d), indicating the formation of a protein corona around PB NPs. The components of the protein corona were determined by iTRAQ-based proteomic analysis. The protein corona of PB NPs contained abundant opsonin proteins, such as complement family members (C3, C5, C9, C4b, Cfh, Cfb, Cfd, Cfi, C1ra, C4bpa, C8a, C8b), immunoglobulin, laminin, fibronectin, C-reactive protein (CRP) and collagen (Supplementary table S2).

### Clearance of PB NPs from mouse tissues

Mouse organs were harvested at different time points after injection of PB NPs to investigate the in vivo clearance of PB NPs. On the 1st day postinjection, the livers and lungs of treated mice presented dark colors due to the high accumulation of PB NPs, while this dark color gradually faded with time (Supplementary Fig. S8). On the 60th day after injection, the livers and lungs of the treated mice presented similar color to that of untreated mice. The tissue clearance of PB NPs was studied by detecting the changes in PB NP-derived Fe content in the tissues, which was calculated by deducting the endogenous tissue Fe content from the total Fe content of PB NP-containing tissues. By deducting the endogenous Fe content, we found that the Fe levels in the hearts, spleens, and kidneys of treated mice were similar to those in untreated mice at each time point, and the accumulation and clearance of PB NPs in these tissues were unobservable (Fig. 3e, 3h, 3i). In contrast, the clearance of PB NPs was observable in the livers and lungs after the deduction of the endogenous tissue Fe content (Fig. 3f, 3g). The PK parameters of Fe from PB NPs in the livers and lungs were further calculated (Supplementary table S3). The AUC_0-∞_, T_1/2_, and MRT of Fe from PB NPs in the liver were evaluated as 9688.83 ± 2534.41 µg/g·day, 20.71 ± 2.21 days, and 29.35 ± 2.54 days, respectively. The AUC_0-∞_, T_1/2_, and MRT of Fe from PB NPs in the lungs of mice were evaluated as 13671.92 ± 4118.49 µg/g·day, 17.33 ± 4.99 days, and 25.48 ± 6.49 days, respectively. The above results suggested that PB NPs undergo slow clearance from the livers and lungs of mice.

***In vitro*** **simulated degradation of PB NPs in simulated body fluid**.

Simulated degradation of PB NPs was investigated in simulated body fluid (SBF) with pH 7.4 (simulating tissue fluid) and pH 4.5 (simulating the acidic environment of intracellular endosomes). After 48 h of incubation, both the color of colloidal PB NPs and the UV‒vis absorption showed attenuation in the two SBFs (Fig. 3j-l), indicating the degradation of PB NPs. XRD and FTIR analyses further proved the degradation of PB NPs (Fig. 3m-n). The diffraction peaks and the vibration of the Fe^II^-CN-Fe^III^ band of PB NPs showed attenuation after incubation (Fig. 3m and 3n), while vibration ascribed to the Fe-O band emerged in the FTIR spectra (Fig. 3n), especially obvious in pH 7.4 SBF, implying the collapse of the lattice of PB NPs. According to the changes in UV‒vis absorptions, XRD and FTIR peaks, and the colors of PB NPs, PB NPs undergo faster degradation in pH 7.4 SBF than in pH 4.5 SBF. Afterward, FeCl_3_ solution was added to the supernatant solutions from the incubated SBFs, and a blue colloid was only generated in the supernatant of pH 7.4 SBF (Supplementary Fig. S9). FTIR analysis showed that the blue colloid presented the specific vibration of PB NPs in the FTIR spectrum (Supplementary Fig. S10), indicating that [Fe(CN)_6_]^3-^ existed in the supernatant of incubated pH 7.4 SBF. This result suggested that in pH 7.4 SBF, the Fe^II^-CN-Fe^III^ bond of PB NPs was broken into [Fe(CN)_6_]^3-^ and Fe^3+^ in the presence of hydroxide ions. Instead of [Fe(CN)_6_]^3-^, CN^-^ was detected in the supernatant of incubated pH 4.5 SBF (Supplementary Fig. S11), it is likely that CN^-^ can be released from Fe^II^-CN-Fe^III^ bonds under acidic conditions [29]. Thus, our results indicated that PB NPs undergo different degradation patterns under different pH conditions.

**Fig. 4:**
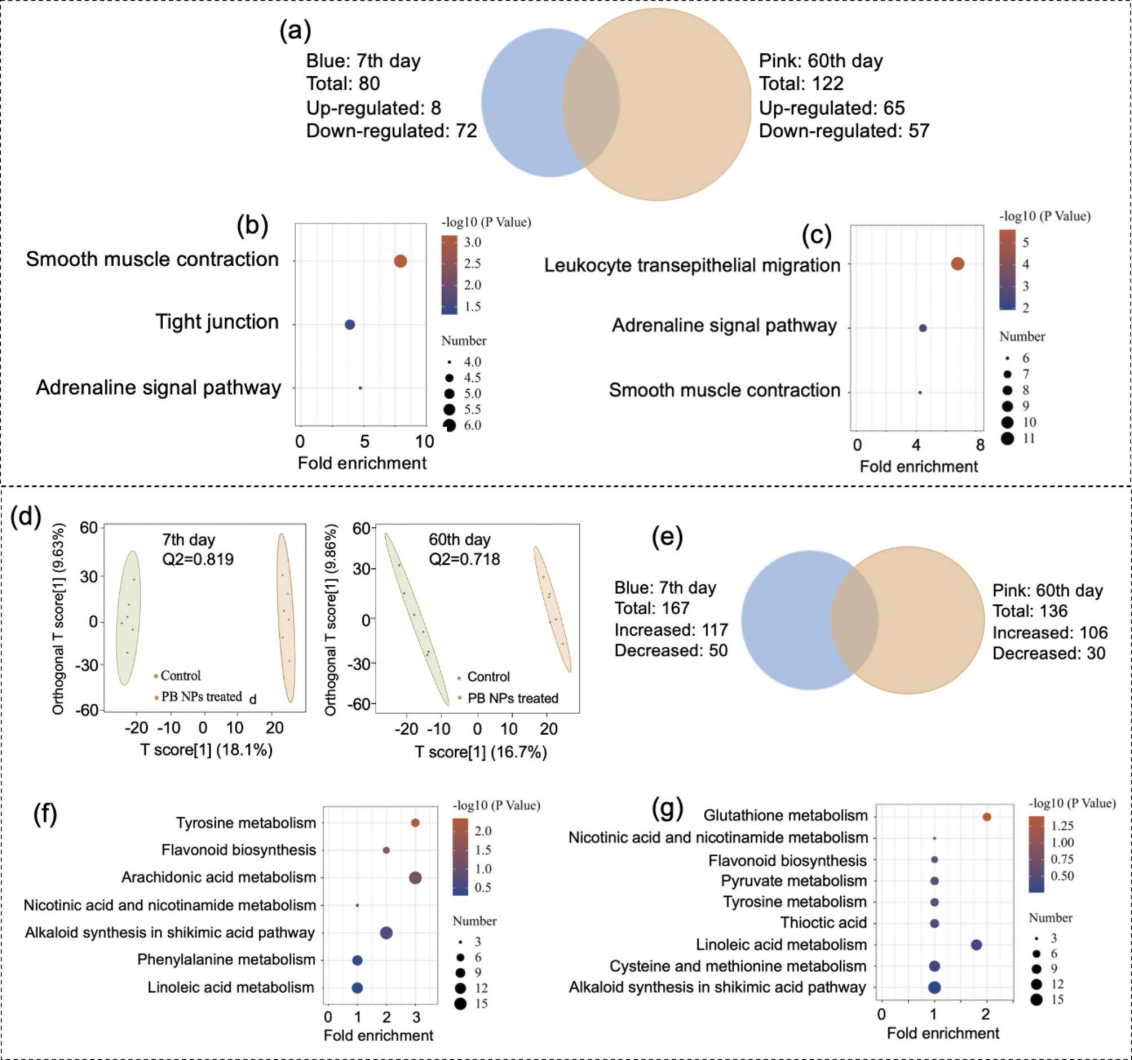
Proteomics and Metabolomics analysis of mouse lungs after i.v. administration of 20 mg/kg PB NPs. **(a)** Venn diagram of the differentially expressed proteins in the lungs. (Blue: 7th day, Pink: 60th day). **(b-c)** KEGG pathway enrichment analysis of differentially expressed proteins in the lungs on the 7th day **(b)** and the 60th day **(c)**. (**d)** OPLS-DA score plot of lung metabolomics. **(e)** Venn diagram of the changed metabolites in the lungs (blue: 7th day, pink: 60th day). **(f-g)** KEGG pathway enrichment analysis of the changed metabolites in the lungs on the 7th day **(f)** and the 60th day **(g)**

### Integrated Proteomics and Metabolomics Analysis of mouse lungs

An integrated omics analysis was used to uncover the physiological response of mouse lung tissues to PB NP exposure. By proteomic analysis, a total of 4676 proteins were identified in lung samples by LC‒MS/MS. Compared with the control group, 80 (8 proteins were upregulated and 72 proteins were downregulated) and 122 (65 proteins were upregulated and 57 proteins were downregulated) differentially expressed proteins were identified in the lung tissues on the 7th and 60th day after PB NP exposure, respectively (Fig. 4a). The PCA score and the volcano plot of proteins identified on the 7th and 60th days are shown in Supplementary Fig. S12. These differentially expressed proteins were analyzed through KEGG pathways (Fig. 4b and 4c), and the proteins related to the main enrichment pathways on the 7th and 60th days are shown in Tables S4 and S5, respectively. Calmodulin (CaM) and myosin light chain (MLC) were downregulated on both the 7th and 60th days after the injection of PB NPs (Table S4-5), and the enriched pathways were smooth muscle contraction and adrenaline signal transduction (Fig. 4b and 4c). Tropomyosin (TPM) and myosin were downregulated on the 7th day (Supplementary table S4), and the enriched pathway was tight junction (Fig. 4b). S100A8/9, LCN2, MMP9, NOX, ITGB2, Vav, Rac2, HK, TF, and ITGAM were upregulated on the 60th day (Supplementary table S5), and these proteins were enriched in the pathway of leukocyte transepithelial migration (Fig. 4c).

In metabolomics analysis, orthogonal partial least squared discrimination analysis (OPLS-DA) showed that the metabolomics data we compared on the 7th and 60th days could be satisfactorily separated (Q2 value > 0.5, as shown in Fig. 4d). A total of 167 (117 metabolites increased and 50 metabolites decreased) and 136 (106 metabolites increased and 30 metabolites decreased) significantly changed metabolites were identified in mouse lungs on the 7th and 60th day post injection of PB NPs, respectively (Fig. 4e). The volcano plot of metabolites identified and hierarchical clustering heatmap of identified differential expression of metabolites (DEMs) in the lungs are shown in Supplementary Fig. S13. Increasing levels of prostaglandin (PGE2, PGA2 and PGJ2), 13-OxoODE (13-keto-9Z,11E-octadecadienoic acid), 4-maleylacetoacetic acid, 2-hydroxybutyric acid, 20-hydroxycholesterol, and α-phocaecholic acid were found in the lung tissues on the 7th day (Supplementary table S6), and the KEGG pathways of shikimic acid, linoleic acid, and arachidonic acid metabolism were enriched (Fig. 4f). The levels of glutathione and anthraniloyl-CoA increased on the 60th day (Supplementary table S7), and the enriched KEGG pathways were glutathione metabolism, pyruvate metabolism, lipoic acid metabolism, and cysteine and methionine metabolism (Fig. 4g). The levels of maleic acid and 3-methylfumaryl-CoA increased on both the 7th and 60th days (Supplementary table S6-7), and the enriched KEGG pathways were the shikimic acid pathway, alkaloid synthesis, tyrosine metabolism, flavonoid biosynthesis, nicotinic acid and nicotinamide metabolism, and linoleic acid metabolism (Fig. 4f and 4g).

**Fig. 5:**
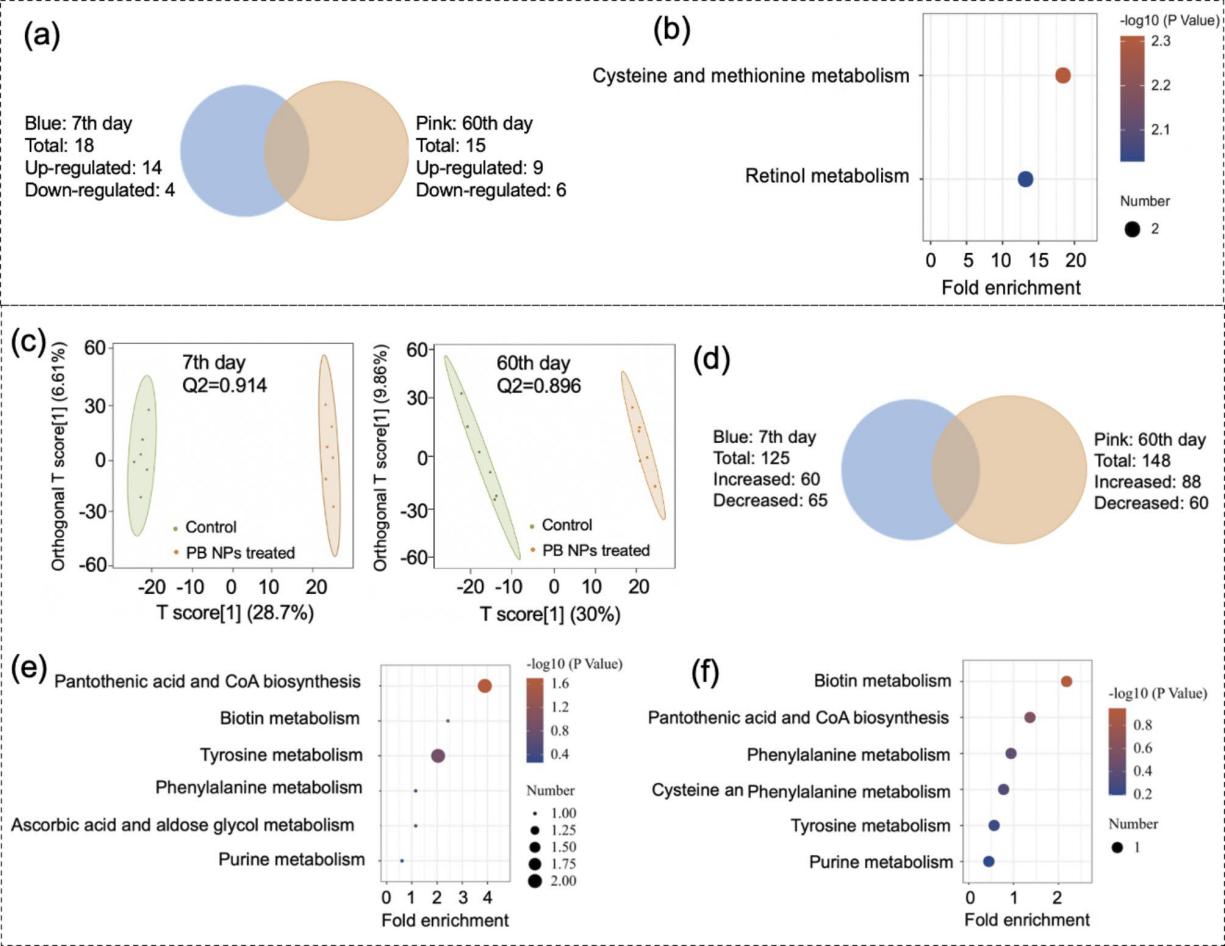
Proteomics and metabolomics analysis of mouse livers after i.v. administration of 20 mg/kg PB NPs. **(a)** Venn diagram of differentially expressed proteins in the liver. (blue: 7th day, pink: 60th day) **(b)** KEGG pathway enrichment analysis of differentially expressed proteins in the livers on the 7th day. (**c**) OPLS-DA score plot of the liver metabolome. **(d)** Venn diagram of changed metabolites in the livers (blue: 7th day, pink: 60th day). **(e-f)** KEGG pathway enrichment analysis of the changed metabolites in the livers on the 7th day **(e)** and the 60th day **(f)**

### Integrated Proteomics and Metabolomics Analysis of mouse livers

We also used integrated omics analysis to uncover the physiological response of mouse livers to the exposure of PB NPs. By proteomics analysis, a total of 5059 proteins were identified in the liver samples by LC‒MS/MS. 18 (14 proteins were upregulated and 4 proteins were downregulated) and 15 (9 proteins were upregulated and 6 proteins were downregulated) differentially expressed proteins were identified in the liver tissues on the 7th and 60th days after PB NPs exposure, respectively (Fig. 5a). The PCA score and the volcano plot of proteins identified on the 7th and 60th days are shown in Supplementary Fig. S14. These differentially expressed proteins were analyzed through the KEGG database, and the proteins related to the main enrichment pathways on the 7th and 60th days are shown in Supplementary tables S8 and S9, respectively. Lactate dehydrogenase (LDH) and cysteine dioxygenase 1 (CDO1) were upregulated in mouse livers on the 7th day after PB NPs exposure (Supplementary table S8), and the enriched KEGG pathways were cysteine and methionine metabolism (Fig. 5b). CYP2A5 and CYP2A12, two members of the cytochrome P450 (CYP) subfamily, were significantly upregulated in mouse livers on the 7th day after PB NPs exposure (Fig. 5b), and the enriched KEGG pathway was the retinol metabolic pathway. We also found that S100A8/9, which is related to the inflammatory response, was upregulated on the 60th day after PB NPs exposure (Supplementary table S9).

In metabolomics analysis (Q2 value for OPLS-DA > 0.5, as shown in Fig. 5c), 125 (60 metabolites increased and 65 metabolites decreased) and 148 (88 metabolites increased and 60 metabolites decreased) significantly changed metabolites were screened on the 7th and 60th days after PB NP exposure (Fig. 5d). The volcano plot of metabolites identified and hierarchical clustering heatmap of identified DEMs in the lungs are shown in Supplementary Fig. S15. The metabolites enriched in the KEGG pathways are shown in Supplementary Tables S10 and S11. The results showed that the levels of d-dethiobiotin, d-discadenine, and 2-hydroxylamino-4,6-dinitrotoluene were increased, and the levels of dephospho-CoA, 4-(4-deoxy-α-D-gluc-4-enuronosyl)-D-galacturonate, 2-amino-2-deoxyisochorismate, and m-cresol were decreased on both the 7th and 60th days after PB NPs exposure. Based on the level changes of the above metabolites, KEGG pathways of purine metabolism, pantothenic acid and CoA biosynthesis, biotin metabolism, tyrosine metabolism, and phenylalanine metabolism were enriched (Fig. 5e and 5f). The levels of D-4’-phosphopantothenate, phenol, 5-hydroxyisourate, and prontosil were increased, the level of N1-(5-phospho-D-ribosyl)-AMP was decreased on the 7th day after PB NPs exposure, and the KEGG pathway of ascorbic acid and aldose glycol metabolism was enriched (Fig. 5e). On the 60th day after PB NPs exposure, 9,10-epoxy-18-hydroxystearate exhibited an increasing level, 5-methylthio-D-ribose, dTDP-D-glucuronate, and (Z)-4-(2-hydroxy-5-sulfonatophenyl)-2-oxo-3-butenoate exhibited a decreasing level, and the cysteine and methionine metabolism pathways were enriched (Fig. 5f).

### Validation of the protein expression of S100A9

Omics analysis revealed that S100A8 and S100A9 were upregulated in the liver and lung tissues of mice on the 60th day after PB NPs exposure. S100A8 and S100A9 are biomarkers of inflammation in the clinic [33]. Here, we further validated the change in S100A9 by western blotting. As shown in Supplementary Fig. S16, S100A9 was upregulated in the liver and lung tissues of mice on the 60th day after i.v. administration of PB NPs (20 mg/kg).

## Discussion

In our study, the fate and potential risks of PB NPs in mice after i.v. administration was evaluated via an integrated toxicology, pharmacokinetics, proteomics, and metabolomics methodology. General toxicological studies showed that a relatively high dose (20 mg/kg) of PB NPs resulted in a transient loss of appetite and body weight in mice in the initial 2 days, but it did not induce apparent acute or subacute toxicities. In mouse bodies, PB NPs cleared quickly from blood after i.v. administration and mainly accumulated in the livers and lungs due to the formation of the protein corona, in which abundant opsonin proteins were identified. Opsonin proteins can activate the complement system and perform opsonin functions, leading to increased phagocytosis by phagocytes [26–28]. The protein corona-mediated opsonin effect is one of the main factors that can affect the biodistribution of NPs [27, 30]. In our study, a protein corona containing opsonin proteins can account for the fast clearance of PB NPs in the blood and the high accumulation of PB NPs in mouse livers and lungs enriched with phagocytes. Additionally, we found that PB NPs underwent slow clearance from the liver and lungs of mice. Meanwhile, we also simulated the degradation of PB NPs in SBF with pH 7.4 and pH 4.5 and found that PB NPs underwent the collapse of the lattice in the two SBFs. In pH 7.4 SBF, [Fe(CN)_6_]^3−^ was released slowly from the PB NP lattice, while CN^−^ was further released in pH 4.5 SBF, these results showed us the possible degradation patterns of PB NPs in the livers and lungs of mice. Since physiological changes at the molecular level are difficult to detect by general toxicological evaluations, we analyzed PB NP-exposed liver and lung tissues from mice (treated with PB NPs at 20 mg/kg) by integrated proteomics and metabolomics analysis and investigated whether PB NPs exposure would affect the physiological functions of mouse liver and lung tissues at the molecular level.

**Fig. 6:**
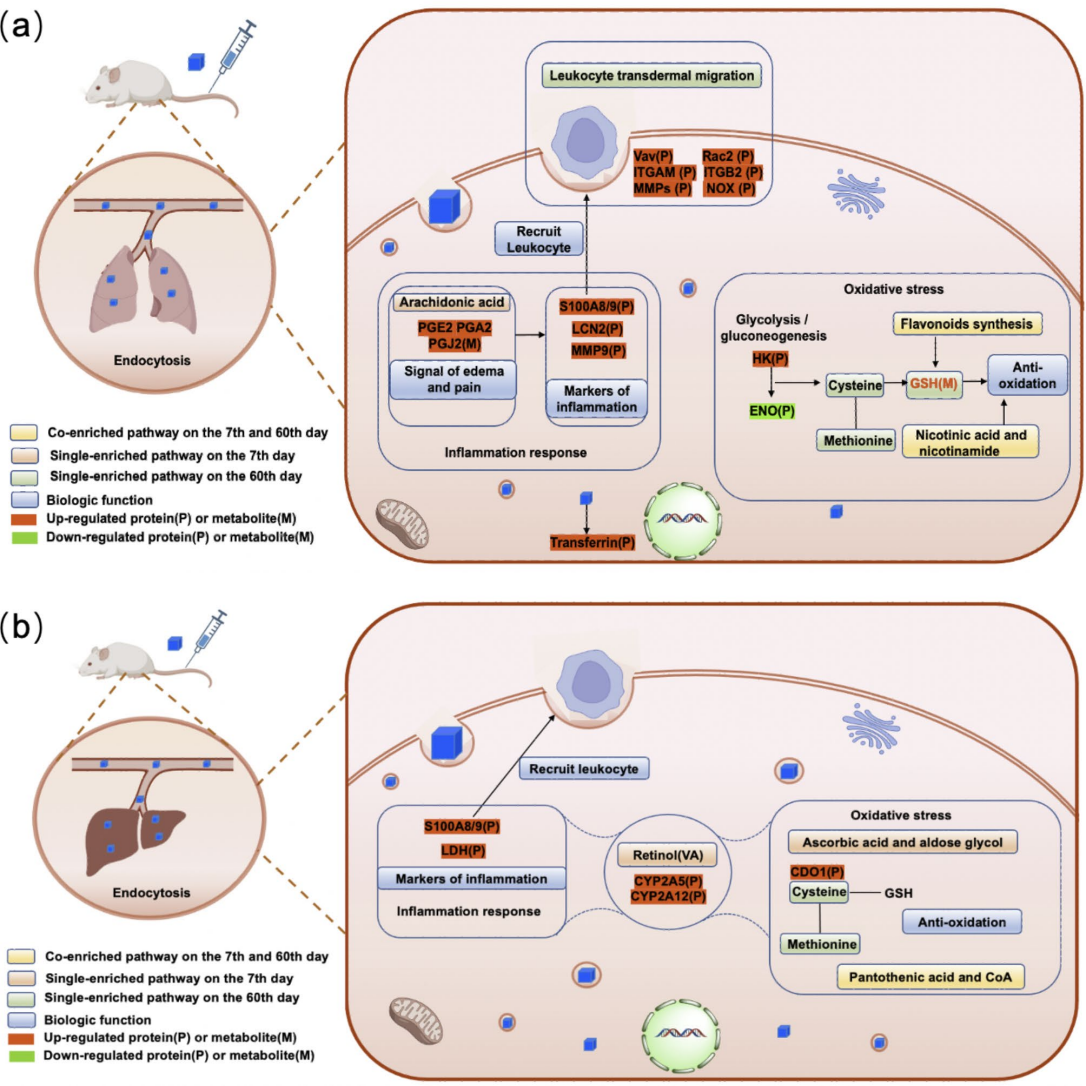
Scheme of major biological pathways responding to PB NPs exposure. (**a**) Scheme of major biological pathways responding to PB NPs exposure in mouse lung tissues. (**b**) Scheme of major biological pathways responding to PB NPs exposure in mouse liver tissues

The inflammatory response is an obvious response of lung tissues to PB NPs exposure (Fig. 6a). Metabolomics analysis revealed that the levels of prostaglandins (PGE2, PGA2 and PGJ2) in the lungs increased on the 7th day. Prostaglandins play important roles in the arachidonic acid metabolism pathway. Both arachidonic acid and prostaglandins could amplify the signal of edema and pain in tissues, and prostaglandin has chemotaxis on leukocytes and is one of the markers of the inflammatory response [31, 32]. The results indicated that an inflammatory reaction occurred in the lung tissues. Proteomics analysis showed that Vav, Rac, ITGAM, ITGB2, S100A8/9, LCN2 and matrix metalloproteinase (MMP9) were upregulated in lung tissues on the 60th day after PB NPs exposure. These proteins play important roles in leukocyte migration and recruitment and mainly participate in anti-inflammatory activity [33–36]. S100A8/9, LCN2 and MMP9 are highly expressed in various inflammatory diseases and are biomarkers of inflammatory reactions. These results were consistent with a previously described study in which the retention of NPs in tissues induced the upregulation of the inflammatory-related gene S100A8/9 [24].

Additionally, the integrated omics analysis uncovered the oxidative stress of tissue cells in response to PB NPs exposure in the lungs of mice (Fig. 6). Flavonoid biosynthesis and nicotinic acid and nicotinamide metabolism were affected to varying degrees on the 7th and 60th days after PB NPs exposure. Flavonoids can promote the production of glutathione (GSH), thereby protecting the body from oxidative damage [37]. Niacin and nicotinamide can reduce ROS content and alleviate the damage caused by oxidative stress [35, 38]. The phenylalanine metabolism pathway was enriched in the lung tissues on the 7th day after PB NPs exposure. Phenylalanine can increase the activity of antioxidant enzymes and reduce oxidative damage in tissues. Glutathione metabolism, pyruvate metabolism, cysteine, and methionine metabolism (Fig. 4g) were the metabolic pathways that were enriched by KEGG pathway analysis in the lungs on the 60th day after PB NPs exposure. These pathways are mainly related to antioxidation [37, 38]. Notably, we detected an increased level of glutathione (GSH), indicating that antioxidation occurred. By proteomics analysis, we observed the upregulation of NOX and hexokinase (HK) and the downregulation of α-enolase (ENO1) on the 60th day. These results further confirmed the occurrence of intracellular oxidative stress [39, 40]. In addition, transferrin was upregulated on the 60th day, which reflected the enhanced iron metabolism due to the high iron concentration resulting from the degradation of PB NPs. It has been reported that iron overload in tissues can cause tissue or cell damage by inducing ROS overproduction or catalyzing lipid peroxidation [41]. The above results suggest that oxidative stress reactions occurred in the lung tissues on the 7th and 60th days after PB NPs exposure, and the oxidative stress reaction may continue in the lung tissues within 60 days after PB NP exposure.

For the liver tissues, LDH was found to be upregulated on the 7th day after PB NPs exposure by proteomics analysis. LDH is an inflammatory marker [42, 43], and an increased LDH level often indicates the occurrence of inflammatory reactions. CYP2A5 and CYP2A12 were significantly upregulated in liver tissues on the 7th day after PB NPs exposure (Fig. 6b). The CYP subfamily mainly exists in the endoplasmic reticulum of hepatocytes and participates in intracellular metabolism. It has been reported that upregulation of CYP2A5 occurs in the state of hepatitis and liver injury [44–46]. S100A8/9 was upregulated in the liver on the 60th day after PB NPs exposure, and a similar result was found in the proteomics analysis of lung tissues. The results indicated that the long-term exposure of liver tissues to PB NPs leads to a local inflammatory reaction.

The omics analysis results also uncovered the oxidative stress of tissue cells in response to PB NPs exposure in the liver (Fig. 6b). It has been reported that upregulation of CYP2A5 also occurs in the state of intracellular oxidative stress [46]. The activation of the ascorbic acid pathway promotes GSH metabolism, can eliminate excessive free radicals in the body, and has significant antioxidant capacity. It can also activate macrophages, inhibit the secretion of proinflammatory factors in macrophages, and has anti-inflammatory and immune regulatory functions [47]. The change in the ascorbic acid pathway indicated that PB NPs may cause oxidative stress in the liver tissue at the initial stage of accumulation. Cysteine and methionine metabolism play a role in antioxidation by participating in the synthesis of glutathione [37]. Furthermore, several intracellular metabolic pathways, such as purine metabolism, pantothenic acid and CoA biosynthesis, biotin metabolism, tyrosine metabolism, and phenylalanine, were affected to varying degrees on the 7th and 60th days due to the accumulation of PB NPs. These metabolic pathways mainly participate in the synthesis of glutathione to indirectly regulate antioxidants [47, 48], supporting the fact that the accumulation and clearance of PB NPs in the liver may cause oxidative stress.

Overall, the results from omics analysis indicated that the accumulation and clearance of PB NPs in mouse liver and lungs induced changes in various physiological activities, and these changed pathways were mainly related to the inflammatory response and intracellular oxidative stress. These changes may continue in mouse livers and lungs within 60 days after PB NPs exposure. Meanwhile, we compared omics analysis results on the 7th and 60th days and found that antioxidation and anti-inflammatory activities were highly activated on the 60th day to prevent tissue damage (Fig. 6).

### Conclusions

In summary, the fate and potential risks of PB NPs in mice after i.v. administration was evaluated via an integrated toxicology, pharmacokinetics, proteomics, and metabolomics methodology. General toxicological studies showed that a high dose (20 mg/kg) of PB NPs only resulted in a transient loss of appetite and body weight in mice, but it did not induce apparent acute or subacute toxicities. PB NPs cleared quickly from blood and mainly accumulated in the livers and lungs of mice and could be gradually removed from the liver and lung tissues with time. Integrated proteomics and metabolomics analysis revealed that oxidative stress and inflammatory reactions occurred during the accumulation and clearance of PB NPs in the livers and lungs of mice. Collectively, our study provides relatively deep insight into the biosafety of PB NPs, which will provide detailed references and guidance for further clinical application of PB NPs in the future.

## Materials and methods

### Reagents

Tris(hydroxymethyl)aminomethane, trifluoroacetic acid, iodoacetamide, dithiothreitol, trichloroacetic acid, TEAB, acetonitrile, formic acid, and methyl alcohol (chromatographic grade) were purchased from Sigma‒Aldrich. Deionized water (18.2 MΩ cm) was obtained by purification of distilled water with a Milli-Q gradient system (Billerica, MA, USA). BCA kits were purchased from Thermo Fisher. All other reagents (analytical grade) were purchased from Sino Pharm Chemical Reagent Co., Ltd. (Shanghai, China).

### Synthesis and characterization of PB NPs

Citric acid-modified PB NPs were synthesized by a previously reported protocol [25]. Briefly, 0.5 mmol of citric acid (98 mg) was added to 20 mL of FeCl_3_ aqueous solution (1.0 mM), and the mixture solution was slowly added to 20 mL of 1.0 mM K_4_[Fe(CN)_6_] aqueous solution (containing 0.5 mmol of citric acid) under stirring at 60 ℃ for 20 min. The mixture solution was continuously stirred for 30 min at 60 ℃ and then cooled to room temperature. PB NP solution was purified and concentrated using filters (100 kDa MWCO, Millipore). The particle size and zeta potential of PB NPs were determined by a Malvern Zeta sizer ZS90 (Malvern Instruments, UK). The NP morphology was observed by a JEOL-2100 transmission electron microscope (TEM). The optical behaviors, structure, and specific groups of PB NPs were characterized with a UV‒vis spectrometer (UV-1800, Shimadzu), X-ray diffractometer (Empyrean), and fluorescence spectrometer (RF-6000, Shimadzu), respectively.

### Degradation property of PB NPs in simulated body fluids

The degradation property of PB NPs was investigated at 37 ℃ in simulated body fluid (SBF) with pH 7.4 and 4.5. At different time intervals, the incubated PB NPs were collected and characterized by UV‒vis spectrometry, X-ray diffraction (XRD) and Fourier transform infrared spectroscopy (FTIR). To prepare SBF, NaCl (8 g), KCl (0.3 g), NaHCO_3_ (0.35 g), K_2_HPO_4_ (0.228 g), CaCl_2_ (0.277 g), Na_2_SO_4_ (0.07 g), MgCl_2_ (0.3 g) and Tris (6 g) were dissolved in deionized water to 1 L, and then the solution was adjusted to the desired pH for the following experiments.

### Animals

Healthy male ICR mice aged 5–6 weeks (20 ± 2 g) were purchased from the Laboratory Animal Center of Jilin University (Changchun, China). The mice were fed in a stable environment (temperature: 25 °C, humidity: 70%, 12 h light/dark cycle) with free access to water and food. All animal handling procedures were strictly performed according to the operating guidelines approved by the institutional animal care and use committee at Northeast Forestry University.

### General toxicity evaluation

Healthy male mice were randomly divided into 4 groups (n = 8) and treated with saline and three different dosages of PB NPs (5, 10, and 20 mg/kg) via i.v. injection, respectively. After different injections, the behavior and body weight of the mice were monitored. Major organs (heart, liver, lung, spleen, kidney, thymus) were harvested on the 1st, 7th, 14th, and 30th days after injection for the calculation of the organ coefficient. After that, the organs were promptly immersed in 10% formalin solution for at least 24 h. Histopathological changes in tissue sections with hematoxylin and eosin (H&E) staining were observed under an optical microscope (Olympus X71, Japan). Mouse blood was collected on the 1st, 7th, 14th, and 30th days after injection. The examination of hematology analysis (n = 8) was carried out by a blood cell analyzer (HF-3800, China). The evaluation of the serum biochemical parameters (n = 8), including glutamic oxaloacetic transaminase (AST), glutamic-pyruvic transaminase (ALT), alkaline phosphatase (ALP), blood urea nitrogen (BUN) and creatinine (CRE), was carried out by a semiautomatic biochemistry analyzer (HF-800 C, China).

### Fe content assay in samples via ICP‒OES

Blood from mice (n = 5) was collected at 0.083, 0.25, 0.5, 1, 2, 4, 8, 12, and 24 h after a single i.v. injection of 20 mg/kg PB NPs. The main tissues (heart, liver, spleen, lung, and kidney) of mice (n = 5) were collected on the 1st, 7th, 14th, 30th, and 60th day after a single i.v. administration of 20 mg/kg PB NPs. To eliminate the interference of exogenous Fe from blood in the tissues, the mice were perfused with saline (containing 0.1% heparin and 0.01% KNO_3_). Afterward, all the samples were lyophilized and digested with nitric acid/perchloric acid at a volume ratio of 1:1 in polytetrafluoroethylene (PTFE) tubes at 120–160 ℃. The digested products were dissolved in a 2% nitric acid solution to measure the Fe content by inductively coupled plasma‒optical emission spectrometry (ICP‒OES). The concentration range of Fe for a standard curve is 0.01-10 µg/mL.

### Protein corona assay

PB NPs were incubated with fresh mouse plasma at 37 ℃ for 30 min for the formation of a protein corona on the PB NPs surface, and then PB NPs coated with the protein corona were purified by centrifugation. The particle size and zeta potential of PB NPs coated with the protein corona were evaluated. The components of the protein corona on PB NPs were analyzed by iTRAQ analysis.

### Proteomic analysis

Proteomic analysis was performed by the conventional protocol. The sampling times were the 7th and 60th days after injection. The fresh tissue samples (n = 5) were frozen and powdered in liquid nitrogen and then incubated with 4-fold volume pyrolysis buffer (8 M urea, 1% protease inhibitor, and 2 mM EDTA) under sonication. Total protein was collected by centrifugation for 10 min (4 ℃, 12 000 g), and the protein concentration was determined by a BCA kit. After the digestion of total protein, the peptides were desalted and vacuum dried. For TMT/iTRAQ labeling, peptides were dissolved in 0.5 M TEAB and mixed with acetonitrile containing TMT/iTRAQ reagent. The reconstituted peptides were separated into 60 fractions and analyzed by LC‒MS/MS. The above TMT proteomics analysis is supported by Jingjie PTM BioLabs.

### Metabolomic analysis

Metabolomic analysis was performed by the conventional protocol. The sampling times were the 7th and 60th days after injection. Tissue samples (n = 6) were homogenized in 70% methanol solution (-20 °C, containing 1 ppm dichlorophenylalanine). After the homogenate operation, the sample was vibrated and placed on ice for 15 min followed by centrifugation for 10 min (12,000 rpm, 4 °C) to collect the liquid supernatant. After that, the precipitate was reimmersed in ethyl acetate/methanol solution (1:3 by vol), vibrated and placed on ice for 15 min, followed by centrifugation for 10 min (12,000 rpm, 4 ℃) to collect the liquid supernatant. Finally, the mixed supernatant was vacuum dried, ultrasonically dissolved in 70% methanol solution, and centrifuged for 3 min (12,000 rpm, 4 °C) for LC‒MS/MS analysis. The above metabolomic analysis was supported by Metware Biotechnology Co., Ltd.

### Western blot assay

Total protein was extracted from tissues, isolated on a 15% polyacrylamide gel and transferred to nitrocellulose membranes according to the wet transfer method. The membranes were incubated with the corresponding primary antibody (beta-actin, S100A9, 1:1000 dilution) at 4 °C overnight and then incubated with the corresponding secondary antibody (1:2000 dilution). Bound proteins were measured by enhanced chemiluminescence (ECL, Pierce) and analyzed by Image Lab software.

### Statistical analysis

All the data were calculated and analyzed by Microsoft Excel 2016 and GraphPad Prism 8. All results are indicated as the mean ± sd. A t test was used to compare the two groups. One-way ANOVA was used to analyze multiple groups of data. A value of P < 0.05 (*) was considered statistically significant, and a value of P < 0.01 (**) was considered extremely significant. KEGG, NCBI, GO, and UniProt databases were used to annotate, classify, and analyze the changed proteins and metabolites (1.5-fold change and P value < 0.05).

## Electronic supplementary material

Below is the link to the electronic supplementary material.

Supplementary Material 1

## Data Availability

The datasets obtained and/or analyzed during the current study are available from the corresponding author upon reasonable request.
