# Supplementary material for: Uncovering the Fate and Risks of Intravenously Injected Prussian Blue Nanoparticles in mice by an Integrated Methodology of Toxicology, Pharmacokinetics, Proteomics, and Metabolomics
Source: Part Fibre Toxicol. 2023 May 5;20:18. doi: 10.1186/s12989-023-00529-7 (PMC10161560; doi:10.1186/s12989-023-00529-7)
Supplement: Supplementary file 1 — Supplementary Material 1 [file 12989_2023_529_MOESM1_ESM.docx]

**Supplementary Material**

**Uncovering the Fate and Risks of Intravenously injected Prussian Blue Nanoparticles in mice by an Integrated Methodology of Toxicology, Pharmacokinetics, Proteomics, and Metabolomics**

Haijing Qu^1†^, Xing Jin^1†^, Wei Cheng^2^, Dongqi Wu^1^, Boyu Ma^1^, Chenmei Lou^1^, Jian Zheng^1^, Lijia Jing ^1^ *, Xiangdong Xue ^2^ *, Yang Wang^1^ *

^1^ School of Life Science, Northeast Forestry University, Harbin 150040, China.

^2^ School of Pharmacy, Shanghai Frontiers Science Center for Drug Target Identification and Drug Delivery, Shanghai Jiao Tong University, Shanghai 200240, China.

*Corresponding author: [jinglijia@nefu.edu.cn](mailto:jinglijia@nefu.edu.cn) (L. Jing); xuexd@sjtu.edu.cn (X. Xue); ywang1971@hotmail.com (Y. Wang).

^†^ These authors contributed equally to this work.

**Table and Figure Captions**

**Table** **S1.** PK parameters of Fe in mice blood after i.v. administration of 20 mg/kg PB NPs.

| Parameters | AUC_0-∞_ (μg/g∙h) | T_1/2_(h) | MRT(h) |
| --- | --- | --- | --- |
| PB NPs treated group | 1365.89 ± 206.79 | 1.00 ± 0.41 | 1.70 ± 0.44 |

**Table S2.** Identification of the components of protein corona of PB NPs by iTRAQ-based proteomic analysis.

| Protein accession | Protein description | Gene name | M (kDa) | Score | Coverage (%) | |
| --- | --- | --- | --- | --- | --- | --- |
| P01027 | Complement C3 | C3 | 186.48 | 323.31 | | 66.1 |
| P07724 | Serum albumin | Alb | 68.692 | 323.31 | | 83.7 |
| P11276 | Fibronectin | Fn1 | 272.53 | 323.31 | | 38.6 |
| Q921I1 | Serotransferrin | Tf | 76.723 | 323.31 | | 65.4 |
| P28665 | Murinoglobulin-1 | Mug1 | 165.3 | 323.31 | | 52.6 |
| P06909 | Complement factor H | Cfh | 139.14 | 323.31 | | 47.5 |
| P01029 | Complement C4-B | C4b | 192.91 | 323.31 | | 47.1 |
| P08226 | Apolipoprotein E | Apoe | 35.866 | 303.16 | | 45 |
| P28666 | Murinoglobulin-2 | Mug2 | 162.38 | 262.1 | | 40.7 |
| P06728 | Apolipoprotein A-IV | Apoa4 | 45.029 | 244.9 | | 68.6 |
| Q00623 | Apolipoprotein A-I | Apoa1 | 30.615 | 229.23 | | 67 |
| P02088 | Hemoglobin subunit beta-1 | Hbb-b1 | 15.84 | 198.25 | | 93.9 |
| P04186 | Complement factor B | Cfb | 85.004 | 196.63 | | 36.8 |
| P08607 | C4b-binding protein | C4bpa | 51.523 | 176.08 | | 37.1 |
| P97290 | Plasma protease C1 inhibitor | Serping1 | 55.584 | 166.38 | | 31.9 |
| P06684 | Complement C5 | C5 | 188.88 | 156.01 | | 14 |
| P01872 | Ig mu chain C region | Ighm | 49.971 | 149.35 | | 39.4 |
| Q8CG16 | Complement C1r-A subcomponent | C1ra | 80.072 | 146.73 | | 23.8 |
| Q61129 | Complement factor I | Cfi | 67.26 | 137.89 | | 25 |
| P01898 | H-2 class I histocompatibility antigen, Q10 alpha chain | H2-Q10 | 37.251 | 130.08 | | 38.5 |
| Q8CG14 | Complement C1s-A subcomponent | C1sa | 76.857 | 114.47 | | 28.6 |
| E9PV24 | Fibrinogen alpha chain | Fga | 87.428 | 105.51 | | 16.9 |
| P06683 | Complement component C9 | C9 | 62.002 | 101.78 | | 22.4 |
| P01867 | Ig gamma-2B chain C region | Igh-3 | 44.259 | 94.933 | | 36.1 |
| E9Q414 | Apolipoprotein B-100 | Apob | 509.43 | 85.835 | | 3.3 |
| Q8BH35 | Complement component C8 beta chain | C8b | 66.229 | 85.33 | | 22.9 |
| Q8K182 | Complement component C8 alpha chain | C8a | 66.08 | 76.607 | | 20.8 |
| P03953 | Complement factor D | Cfd | 28.057 | 65.602 | | 37.5 |

**Table S3.** The PK parameters of Fe in mice lungs and liver after i.v. injection of PB NPs at 20 mg/kg.

| Parameters | AUC_0-∞_ (μg/g∙day $\cdot day$) | T_1/2_(day) | MRT (day) |
| --- | --- | --- | --- |
| Lung | 13671.92 ± 4118.49 | 17.33 ± 4.99 | 25.48 ± 6.49 |
| Liver | 9688.83 ± 2534.41 | 20.71 ± 2.21 | 29.35 ± 2.54 |

**Table S4.** Differential proteins enriched in KEGG pathways in mice lungs on the 7th day after i.v. injection of PB NPs at 20 mg/kg.

| Protein accession | | Abbreviation | Gene name | | M[kDa] | Regulated | P value |
| --- | --- | --- | --- | --- | --- | --- | --- |
| Q8CI43 | MLC | | Myl6b | 22.749 | | Down | 0.0123389 |
| Q9D6P8 | CaM | | Calml3 | 16.701 | | Down | 0.0300770 |
| P97457 | MLC-2 | | Mylpf | 18.955 | | Down | 0.0036953 |
| P58774 | TPM | | Tpm2 | 32.836 | | Down | 0.0000035 |
| P13541 | Myosin | | Myh3 | 223.79 | | Down | 0.0000047 |
| Q5SX40 | Myosin | | Myh1 | 223.34 | | Down | 0.0000003 |
| P13542 | Myosin | | Myh8 | 222.7 | | Down | 0.0008796 |
| Q5SX39 | Myosin | | Myh4 | 222.86 | | Down | 0.0008628 |

**Table S5.** Differential proteins enriched in KEGG pathways in mice lungs on the 60th day after i.v. injection of 20 mg/kg PB NPs

| Protein accession | | Abbreviation | Gene name | M[kDa] | Regulated | P value |
| --- | --- | --- | --- | --- | --- | --- |
| P31725 | S100A8 | | S100a9 | 13.049 | Up | 0.0000004 |
| P27005 | S100A9 | | S100a8 | 10.294 | Up | 0.0001811 |
| P11672 | LCN2 | | Lcn2 | 22.875 | Up | 0.0035777 |
| P41245 | MMP9 | | Mmp9 | 80.534 | Up | 0.0000187 |
| Q3U6G0 | NOX | | Cybb | 65.305 | Up | 0.0002006 |
| P11835 | ITGB2 | | Itgb2 | 85.025 | Up | 0.0002978 |
| P27870 | Vav | | Vav1 | 98.136 | Up | 0.0003954 |
| Q05144 | Rac2 | | Rac2 | 21.441 | Up | 0.0024165 |
| Q3TRM8 | HK | | Hk3 | 100.1 | Up | 0.0016409 |
| Q921I1 | TF | | Tf | 76.723 | Up | 0.0000016 |
| P05555 | ITGAM | | Itgam | 127.48 | Up | 0.0000222 |
| P09542 | MLC | | Myl3 | 22.421 | Down | 0.0000006 |
| P58774 | TPM | | Tpm2 | 32.836 | Down | 0.0000014 |
| Q9JM83 | CaM | | Calm4 | 16.767 | Down | 0.0009370 |
| Q9D6P8 | CaM | | Calml3 | 16.701 | Down | 0.0021624 |
| P17180 | ENO | | Eno1 | 47.024 | Down | 0.0000595 |

**Table** **S6.** Differential metabolites enriched in KEGG pathways in mice lungs on the 7th day after i.v. injection of 20 mg/kg PB NPs.

| Metabolites | M[g/mol] | VIP | Log2FC | Regulated |
| --- | --- | --- | --- | --- |
| Prostaglandin E2 | 352.2250 | 1.6058 | 1.3860 | up |
| 13-OxoODE(13-Keto-9Z,11E-octadecadienoic acid) | 294.2195 | 1.9277 | 1.2671 | up |
| Maleic acid | 116.0110 | 1.7320 | 1.4465 | up |
| N2-(1-Carboxyethyl)-L-arginine | 246.1328 | 1.8410 | 1.0171 | up |
| Prostaglandin A2 | 334.2144 | 1.6740 | 1.0119 | up |
| PGJ2 [11-oxo-15S-hydroxy-prosta-5Z,9,13E-trien-1-oic acid] | 334.2144 | 1.7280 | 1.0302 | up |
| 20-Hydroxycholesterol | 402.3498 | 1.7100 | 1.2621 | up |
| α-Phocaecholic acid | 424.2825 | 1.6540 | 1.6368 | up |
| 3-Methylfumaryl-CoA | 879.1313 | 1.9100 | 1.3158 | up |
| 4-Maleylacetoacetic acid | 200.0321 | 1.8810 | 1.2229 | up |
| 2-Hydroxybutyric acid | 104.0473 | 1.3500 | -1.3013 | down |
| Chloroxanthin | 556.4644 | 2.0350 | -1.6545 | down |
| 2-Hydroxy-2,4-pentadienoate | 114.0317 | 1.8200 | -1.3683 | down |
| 3-(4-hydroxyphenyl)-1-(2,4,6-trihydroxyphenyl) prop-2-en-1-one | 272.0685 | 1.7980 | -1.2918 | down |
| 2,4-Dihydroxyhept-2-enedioate | 190.0477 | 1.6020 | -1.7326 | down |

**Table S7.** Differential metabolites enriched in KEGG pathways in mice lungs on the 60th day after i.v. injection of 20 mg/kg PB NPs

| Metabolites | M[g/mol] | VIP | Log2FC | Regulated |
| --- | --- | --- | --- | --- |
| Glutathione | 307.0838 | 1.6790 | 1.1954 | up |
| Anthraniloyl-CoA | 886.1523 | 1.8201 | 1.0659 | up |
| Maleic acid | 116.0110 | 1.7510 | 1.5014 | up |
| 3-Methylfumaryl-CoA | 879.1313 | 1.8450 | 1.7789 | up |
| 3-Hydroxy-5-oxohexanoyl-CoA | 895.1626 | 1.5930 | 1.9592 | up |
| 5'-monophosphate-2'-deoxyadenosine | 331.0676 | 1.8080 | 1.3439 | up |
| (R)-Methyl malonyl-CoA | 867.1313 | 1.7600 | 1.2821 | up |
| (R)-S-Lactoylglutathione | 379.1049 | 1.7570 | 1.3968 | up |
| 9,10,13-TriHOME | 330.2406 | 1.8680 | -1.3024 | down |

**Table S8.** Differential proteins enriched in KEGG pathways in mice livers on the 7th day after intravenous i.v. of 20 mg/kg PB NPs

| Protein accession | Abbreviation | Gene name | M(kDa) | Regulated | P value |
| --- | --- | --- | --- | --- | --- |
| P20852 | CYP2A5 | Cyp2a5 | 56.74 | Up | 0.0245 |
| P56593 | CYP2A12 | Cyp2a12 | 56.179 | Up | 0.0090 |
| P16125 | LDH | Ldhb | 36.572 | Up | 0.0384 |
| P60334 | CDO1 | Cdo1 | 23.026 | Up | 0.0061 |

**Table S9.** Differential proteins enriched in KEGG pathways in mice livers on the 60th day after i.v. injection of 20 mg/kg PB NPs.

| Protein accession | Abbreviation | Gene name | M(kDa) | Regulated | P value |
| --- | --- | --- | --- | --- | --- |
| P31725 | S100A9 | S100a9 | 13.049 | Up | 0.000739 |
| P27005 | S100A8 | S100a8 | 10.294 | Up | 0.0206 |

**Table** **S10.** Differential metabolites enriched in KEGG pathways in mice livers on the 7th day after i.v. injection of 20 mg/kg PB NPs

| Metabolites | M(g/mol) | VIP | Log2FC | Regulated |
| --- | --- | --- | --- | --- |
| D-4'-Phosphopantothenate | 299.0770 | 1.6047 | 1.1104 | up |
| Phenol | 94.0421 | 1.4887 | 1.3143 | up |
| Prontosil | 291.0774 | 1.2916 | 1.0955 | up |
| d-Dethiobiotin | 214.1319 | 1.7721 | 1.4412 | up |
| d-Discadenine | 304.1625 | 1.7734 | 1.4608 | up |
| 5-Hydroxyisourate | 184.0234 | 1.7760 | 1.0140 | up |
| 2-Hydroxylamino-4,6-dinitrotoluene | 213.0389 | 1.7672 | 2.0122 | up |
| Dephospho-CoA | 687.1484 | 1.2815 | -1.5066 | down |
| 4-(4-Deoxy-α-D-gluc-4-enuronosyl)-D-galacturonate | 352.0672 | 1.8732 | -5.7922 | down |
| 2-Amino-2-deoxyisochorismate | 225.0633 | 1.7828 | -1.2780 | down |
| N1-(5-Phospho-D-ribosyl)-AMP | 559.0714 | 1.5990 | -1.1748 | down |
| m-Cresol | 108.0576 | 1.7281 | -1.9040 | down |

**Table S11.** Differential metabolites enriched in KEGG pathways in mice livers on the 60th day after i.v. injection of 20 mg/kg PB NPs

| Metabolites | M(g/mol) | VIP | Log2FC | Regulated |
| --- | --- | --- | --- | --- |
| d-Dethiobiotin | 214.1319 | 1.8160 | 1.5194 | up |
| d-Discadenine | 304.1625 | 1.6330 | 1.5236 | up |
| 2-Hydroxylamino-4,6-dinitrotoluene | 213.0389 | 1.6108 | 1.5818 | up |
| 9,10-Epoxy-18-hydroxystearate | 314.2453 | 1.2677 | 1.0176 | up |
| Dephospho-CoA | 687.1484 | 1.0944 | -1.4588 | down |
| 4-(4-Deoxy-α-D-gluc-4-enuronosyl)-D-galacturonate | 352.0672 | 1.4241 | -5.9060 | down |
| 5-Methylthio-D-ribose | 180.0455 | 1.2578 | -1.0091 | down |
| 2-Amino-2-deoxyisochorismate | 225.0633 | 1.8529 | -1.1798 | down |
| Adenine | 135.0546 | 1.2144 | -1.6616 | down |
| m-Cresol | 108.0576 | 1.7085 | -1.4426 | down |
| dTDP-D-glucuronate | 578.0525 | 1.4553 | -1.0592 | down |
| (Z)-4-(2-Hydroxy-5-sulfonatophenyl)-2-oxo-3-butenoate | 271.9982 | 1.6720 | -1.3357 | down |


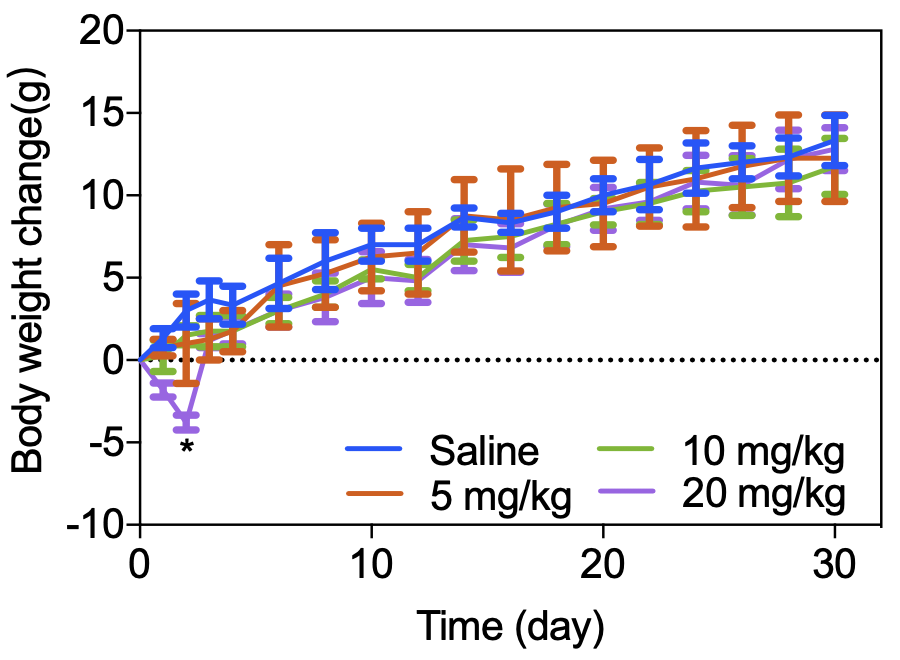


**Fig. S1****.** The body weight growth of mice after i.v. injection of PB NPs at different doses (5, 10 and 20 mg/kg)


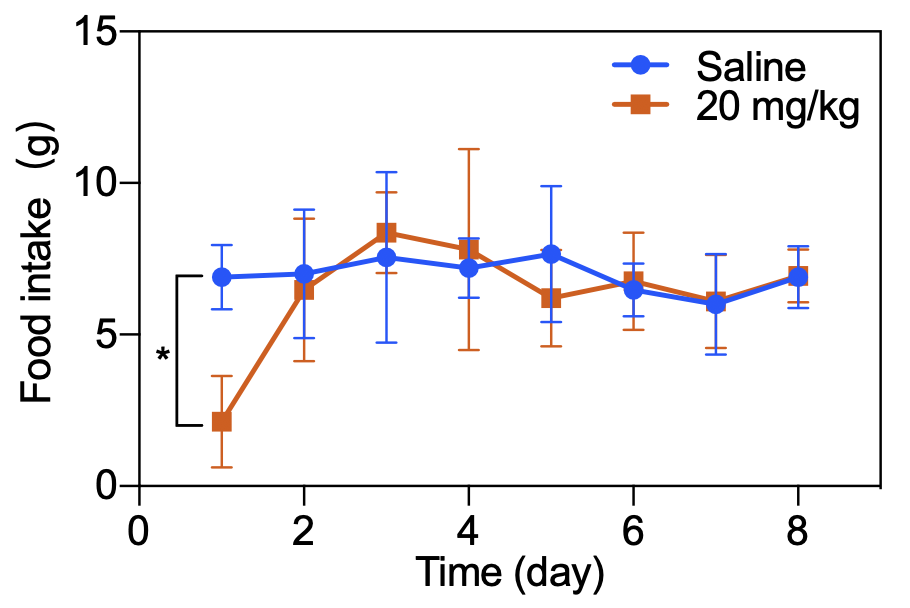


**Fig. S2.** The comparison of food intake between the mice treated with saline and 20 mg/kg PB NPs.


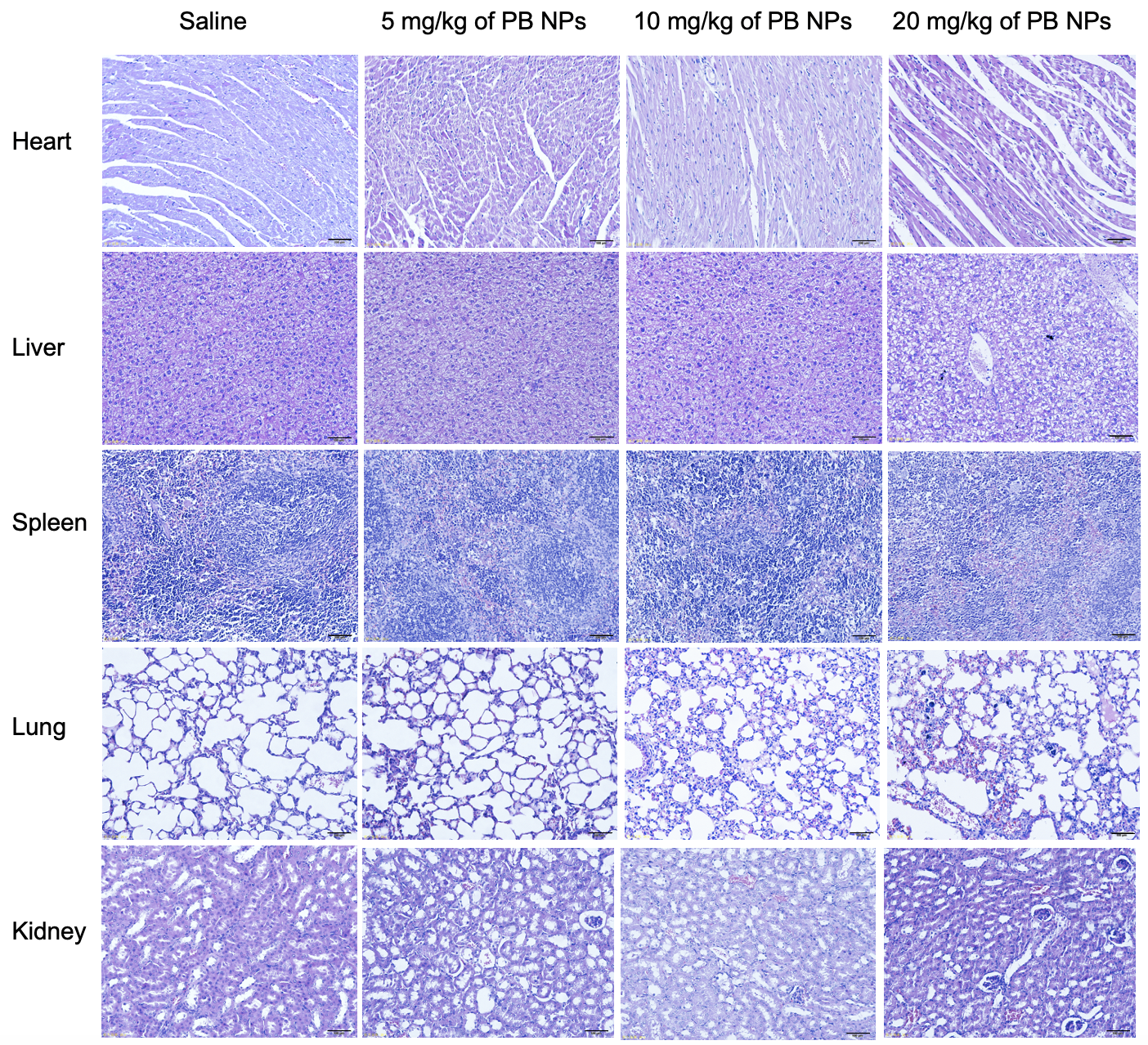


**Fig. S3.** The histopathological sections of mice on the 1st day after i.v. injection of PB NPs at different doses (5, 10 and 20 mg/kg). (Bar: 100 µm)


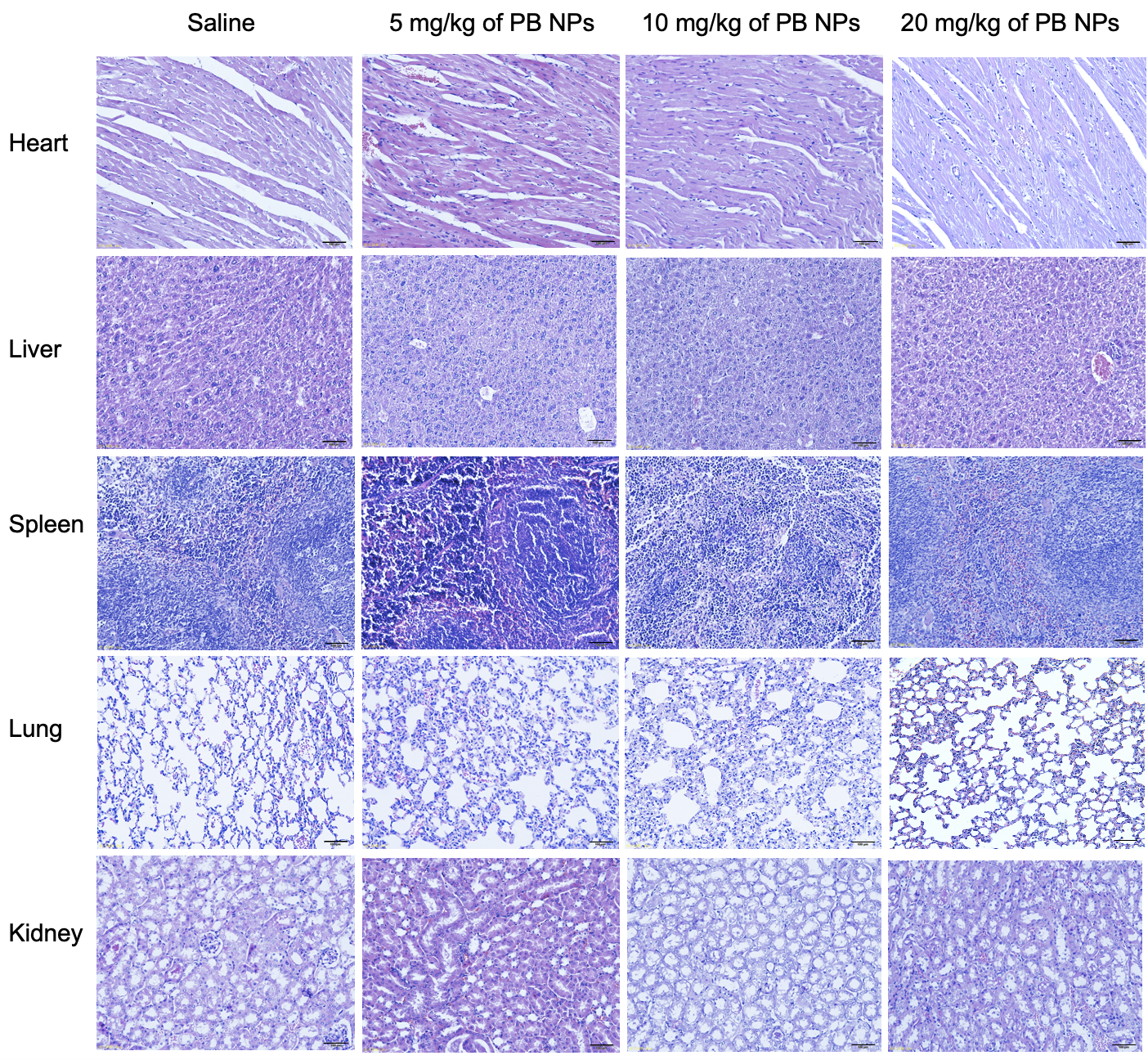


**Fig. S4.** The histopathological sections of mice on the 7th day after i.v. injection of PB NPs at different doses (5, 10 and 20 mg/kg) (Bar: 100 µm)


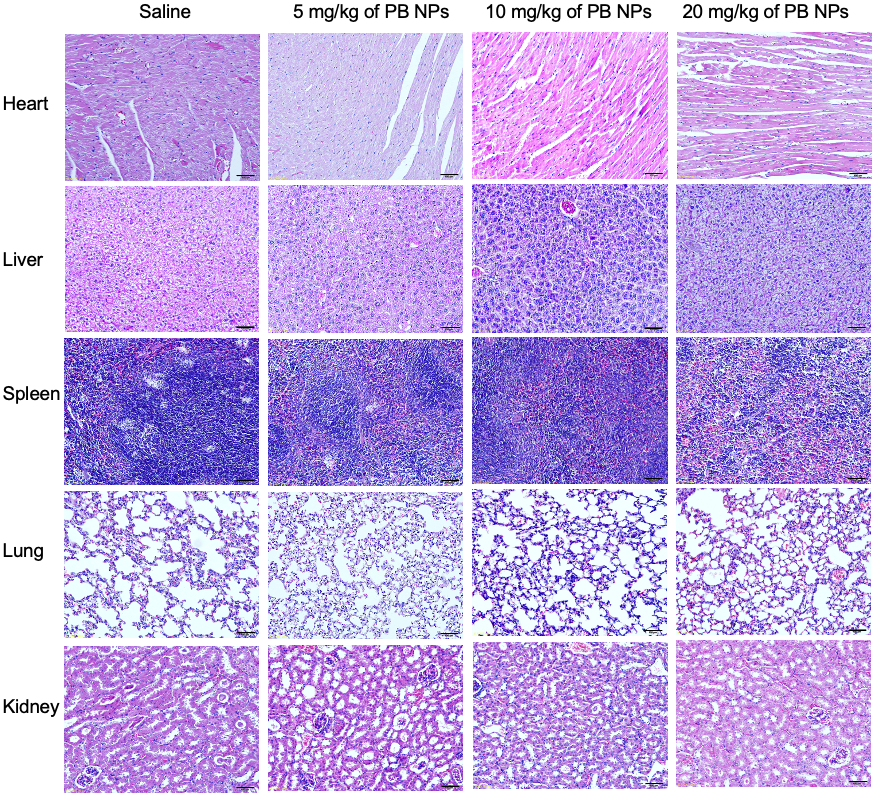


**Fig. S5.** The histopathological sections of mice on the 14th day after i.v. injection of PB NPs at different doses (5, 10 and 20 mg/kg) (Bar: 100 µm)


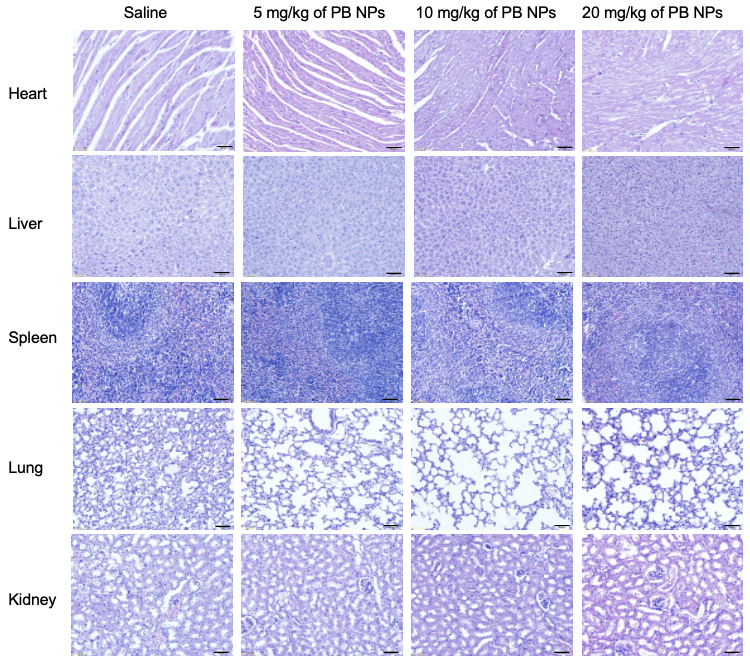


**Fig. S6.** The histopathological sections of mice on the 30th day after i.v. injection of PB NPs at different doses (5, 10 and 20 mg/kg) (Bar: 100 µm)


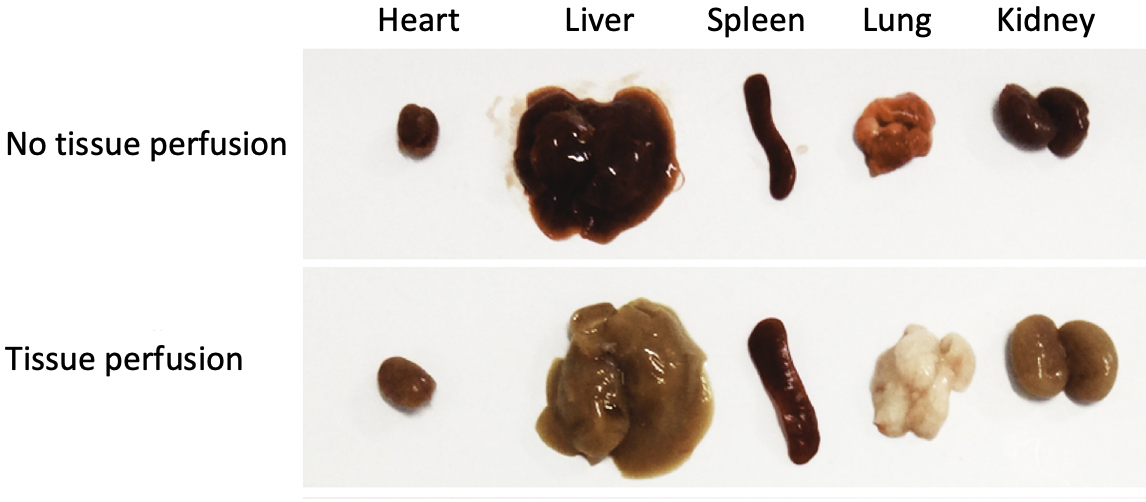


**Fig. S7.** The appearance of mice tissues before and after perfusion.


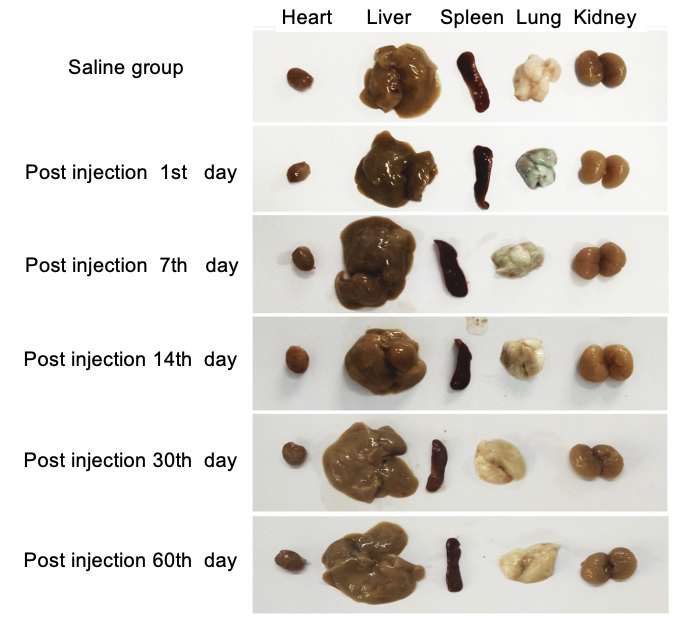


**Fig. S8.** The appearance of mice tissues at different time points after PB BPs injection with perfusion.


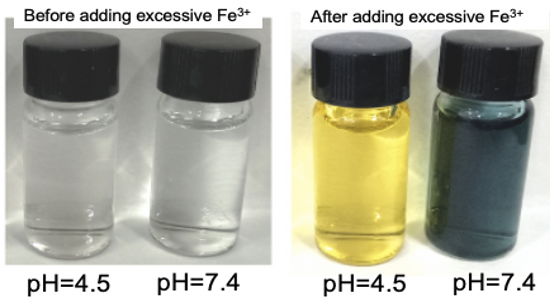


**Fig. S9.** The color change of supernatants in different incubated SBFs after adding excessive iron trichloride.

**Fig. S10.** The FTIR spectrum of precipitates resulted by adding excessive Fe^3+^ to the supernate of pH 7.4 SBF incubating with PB NPs.


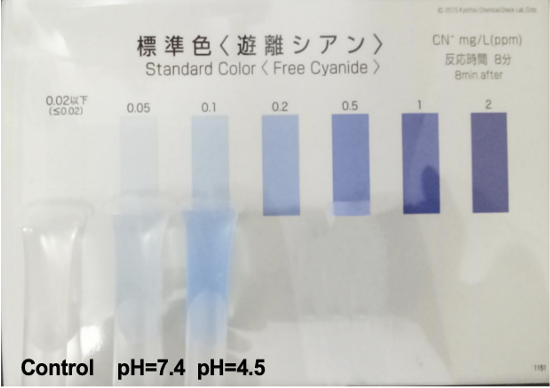


**Fig. S11.** The detection of CN^-^ in the supernate of pH 4.5 and pH 7.4 SBF incubating with PB NPs, respectively.


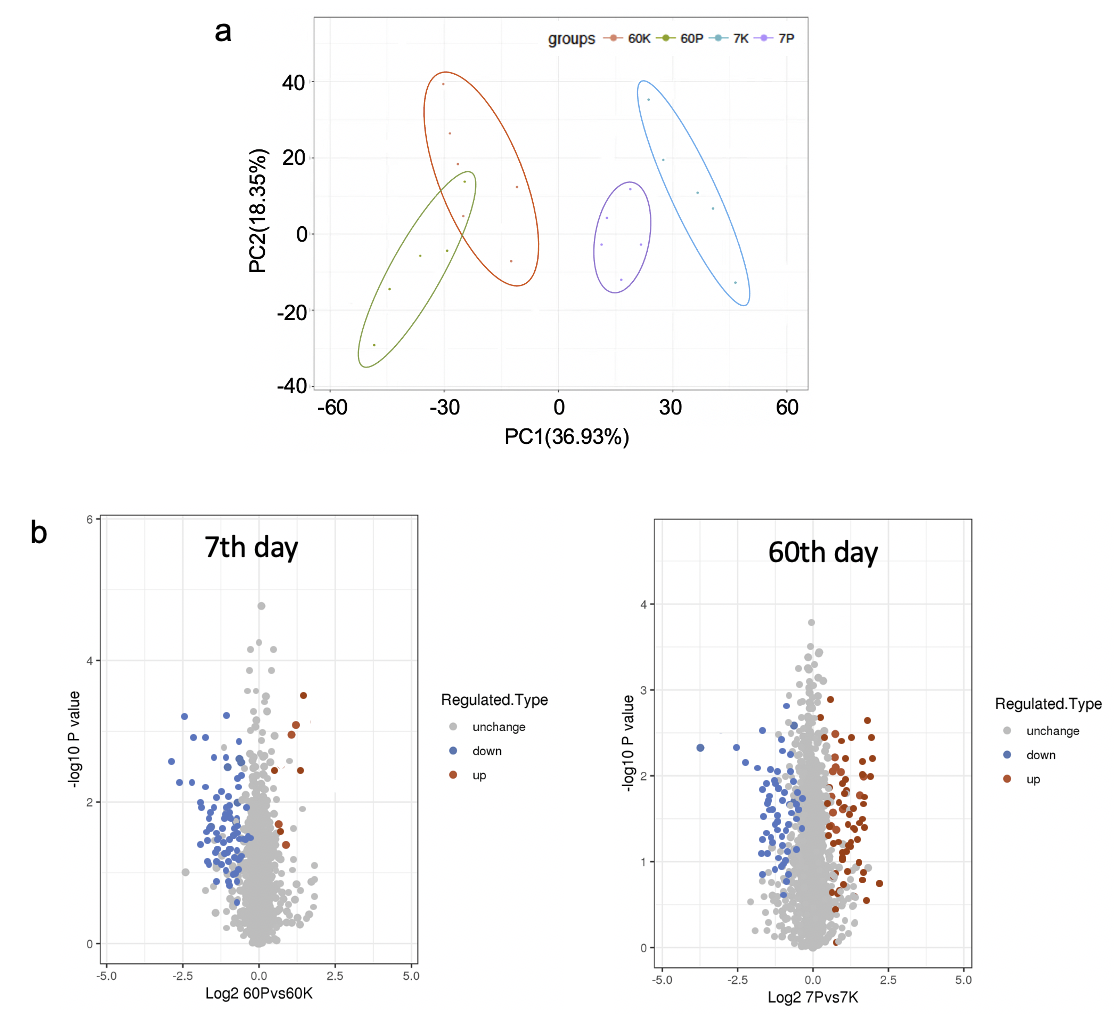


**Fig. S12.** The PCA score **(a)** and the volcano plot **(b)** of proteins identified in the lungs after PB NPs exposure.


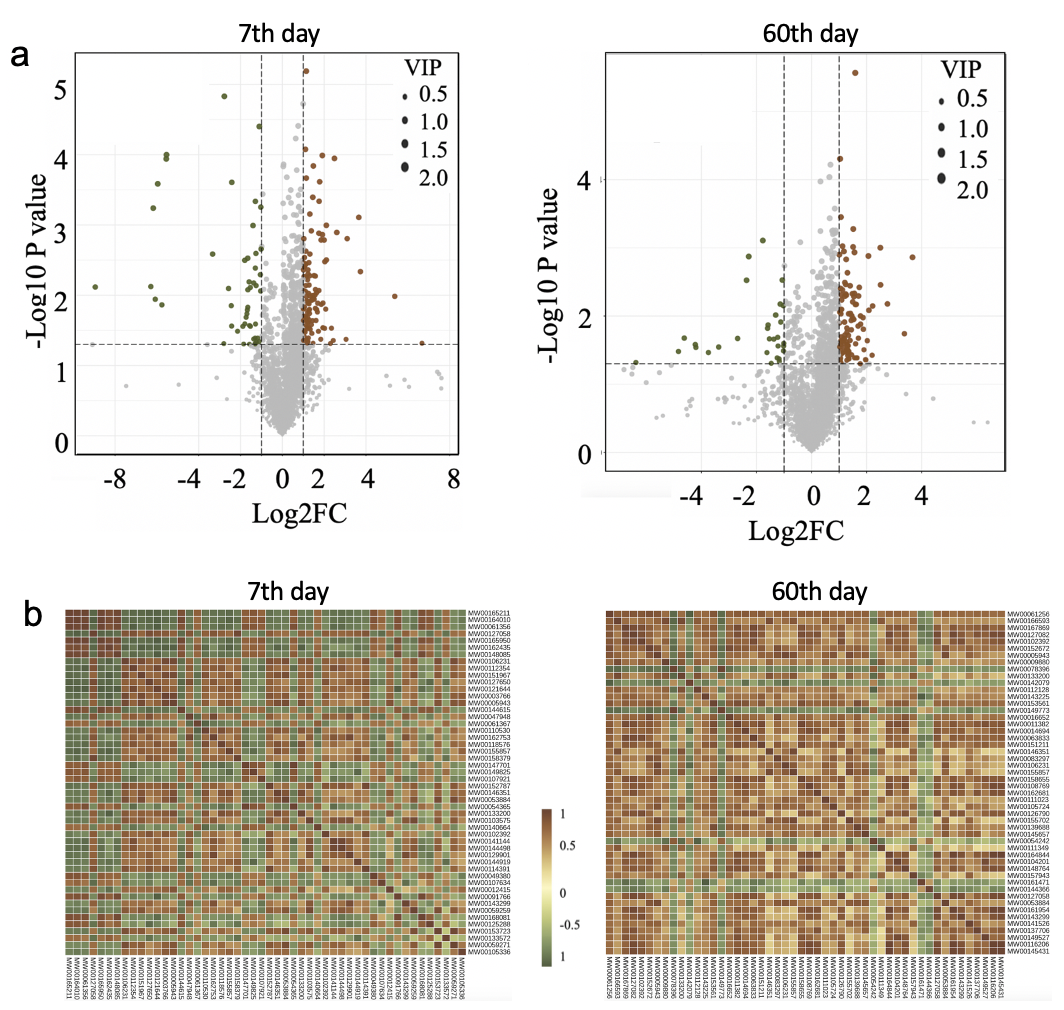


**Fig. S13.** **(a)** The volcano plot of metabolites identified. **(b)** Hierarchical clustering heatmap of identified DEMs in the lungs after PB NPs exposure.


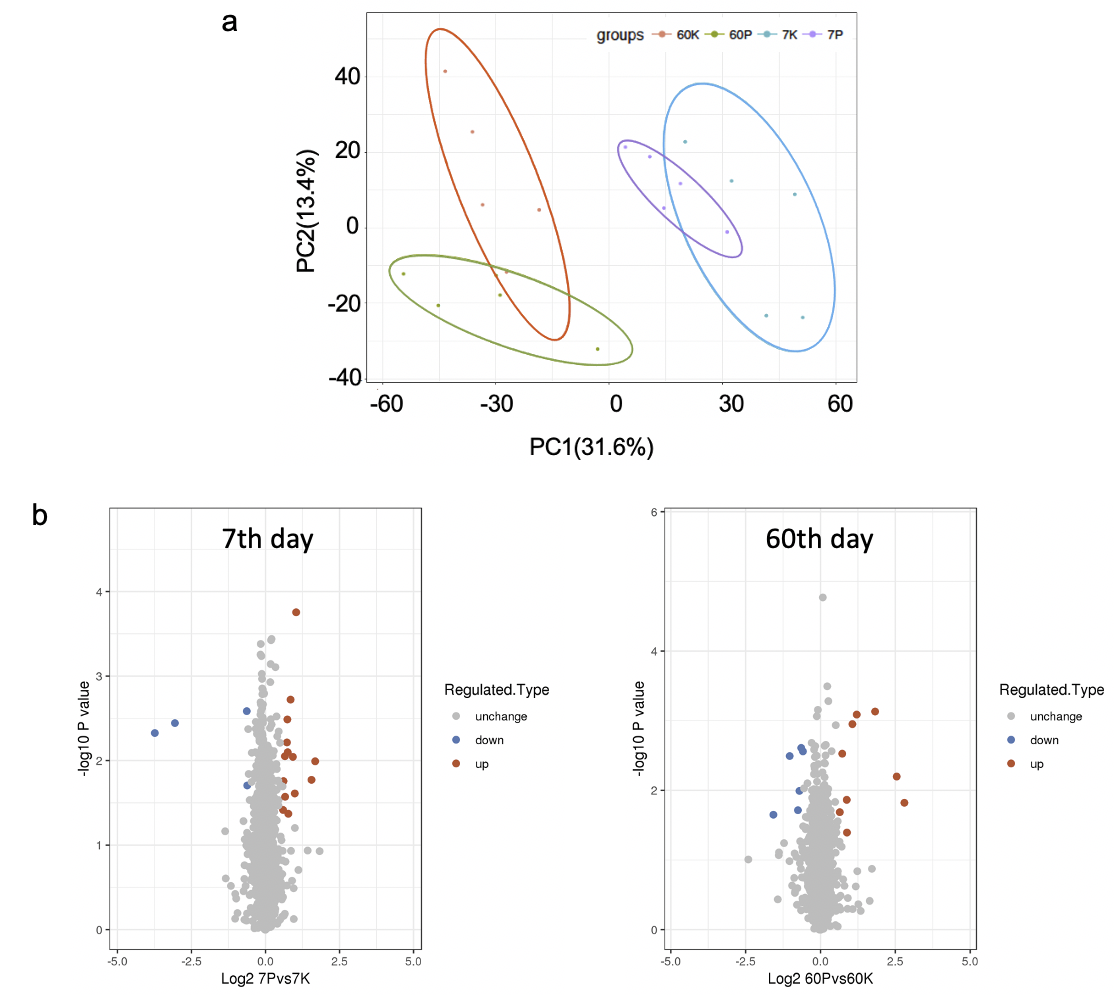


**Fig. S14.** The PCA score **(a)** and the volcano plot **(b)** of proteins identified in the livers of mice after PB NPs exposure.


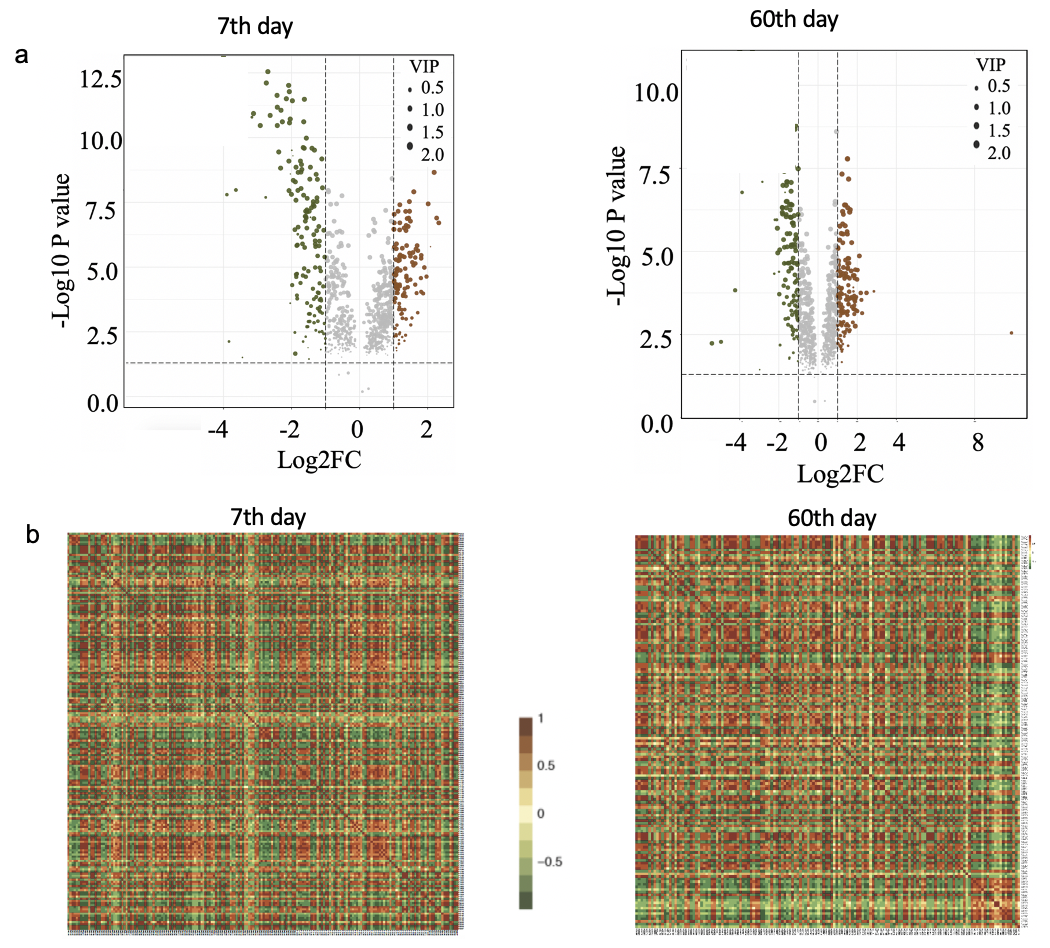


**Fig. S15.** (**a**) The volcano plot of metabolites identified. (**b**) Hierarchical clustering heatmap of identified DEMs in the livers of mice after PB NPs exposure.


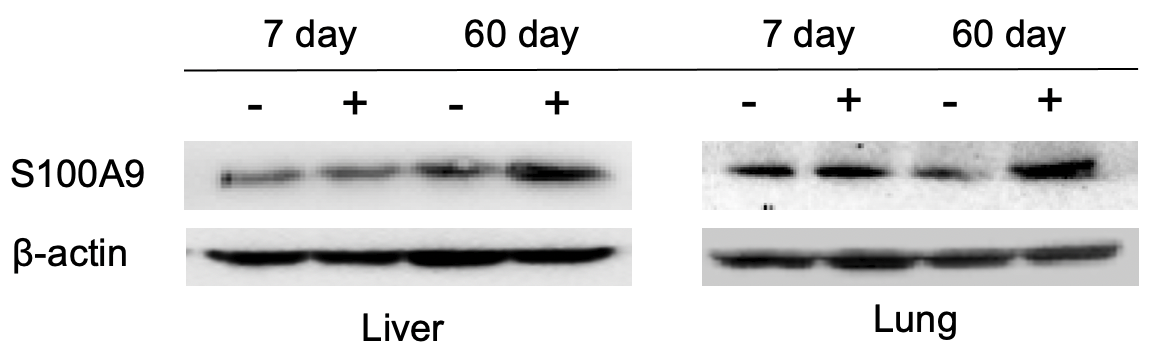


**Fig. S16.** The expression of S100A9 in liver and lung tissues on the 7th and 60th day after PB NPs exposure 20 mg/kg.
